# Balloon catheter plus concurrent vaginal misoprostol versus vaginal misoprostol alone for cervical ripening in induction of labor: A systematic review and an individual participant data meta‐analysis of randomized controlled trials

**DOI:** 10.1002/ijgo.70949

**Published:** 2026-03-12

**Authors:** Malitha Patabendige, Fei Chan, Zainab Al‐Ibraheemi, Davies K. Kibii, Alfred O. Osoti, Philip Ayieko, Tarakeswari Surapaneni, Marino Romero Ramirez, Angeles Vives Argilagos, Sidra Gilani, Syeda B. Mazhar, Daniel L. Rolnik, Ben W. Mol, Wentao Li

**Affiliations:** ^1^ Department of Obstetrics and Gynecology Monash University Melbourne Victoria Australia; ^2^ Obstetrics and Gynecology Unit St John of God Public Hospital Midland Western Australia Australia; ^3^ Department of Obstetrics and Gynecology Mount Sinai West Hospital New York New York USA; ^4^ Department of Obstetrics and Gynecology University of Nairobi Nairobi Kenya; ^5^ Department of Global Health University of Washington Seattle Washington USA; ^6^ Department of Infectious Disease Epidemiology London School of Tropical Medicine and Hygiene London UK; ^7^ Department of Obstetrics, and Clinical Research Unit Fernandez Hospital Hyderabad India; ^8^ Department of Obstetrics and Gynecology Consorci Sanitari de Terrassa Spain; ^9^ Department of Obstetrics and Gynecology, MCH Center Pakistan Institute of Medical Sciences Islamabad Pakistan; ^10^ National Perinatal Epidemiology and Statistics Unit (NPESU), Center for Big Data Research in Health, and School of Clinical Medicine, Faculty of Medicine University of New South Wales Sydney New South Wales Australia

**Keywords:** balloon plus misoprostol, cervical ripening, combined methods, individual participant data, induction of labor, IPD, meta‐analysis, misoprostol, randomized trials, TRACT, trustworthiness

## Abstract

**Background:**

Cervical ripening in labor induction using a combination of methods is gaining popularity, but the effectiveness and safety of this approach are not clear. The assessment of study trustworthiness has recently been raised for evidence synthesis.

**Objective:**

To compare the effectiveness, perinatal and maternal safety of cervical ripening in the induction of labor (IOL) using a balloon catheter with concurrent low‐dose vaginal misoprostol (combined group) versus low‐dose vaginal misoprostol alone.

**Search Strategy:**

MEDLINE, Embase, Emcare, Scopus, Cochrane Library, WHO ICTRP and clinicaltrials.gov.

**Selection Criteria:**

Randomized controlled trials (RCTs), viable singleton gestation, no language restrictions, published and unpublished data.

**Data Collection and Analysis:**

A systematic search, screening for trustworthiness and study quality, and an individual participant data (IPD) meta‐analysis were conducted.

**Main Results:**

A total of eight of 22 (36.4%) trials provided individual participant data, of which three (604 participants) were excluded due to trustworthiness concerns after data checking. A total of 13 of 22 trials (59.1%) were identified as “not meeting trustworthiness criteria.” This individual participant data meta‐analysis included five trials (649 participants): two had prospective, another two had retrospective trial registrations, and one trial was unregistered. Vaginal delivery rate (adjusted odds ratio [aOR] 0.97, 95% confidence interval [CI]: 0.30; 3.12, very low certainty of evidence as downgraded due to imprecision, inconsistency and data completeness), composite adverse perinatal (aOR 0.88, 95% CI: 0.62; 1.24, very low certainty of evidence as downgraded due to imprecision, inconsistency and data completeness) and maternal outcomes (aOR 0.70, 95% CI: 0.33; 1.50, very low certainty of evidence as downgraded due to imprecision, inconsistency and data completeness) were comparable between the two groups in the individual participant data meta‐analysis. Women in the combined group reported significantly less uterine hyperstimulation (aOR 0.20, 95% CI: 0.10; 0.41) and less meconium‐stained liquor (aOR 0.23, 95% CI 0.07; 0.77). Based on aggregate data from eight trials meeting trustworthiness criteria (IPD and non‐IPD), combined methods had an OR of 1.07 (95% CI: 0.68; 1.68) for vaginal delivery. In comparison, data from 13 trials not meeting trustworthiness criteria (IPD and non‐IPD) showed an OR of 1.25 (95% CI: 0.88; 1.77) for vaginal birth.

**Conclusions:**

Based on trustworthy data, the current evidence does not suggest that one method is superior to the other for the effectiveness of the combined balloon catheter with concurrent low‐dose vaginal misoprostol versus low‐dose vaginal misoprostol alone. At present, we are uncertain about the safety of using the combined method due to low data retrieval and trustworthiness concerns among the underlying trials. The results could slightly favor the combined method due to reduced hyperstimulation and meconium‐stained liquor.

## 1 INTRODUCTION

### 1.1 Background

Induction of labor (IOL) is indicated when the benefits of delivery outweigh the maternal or perinatal risks of continuing pregnancy.^1^ Methods for cervical priming and IOL include mechanical, pharmacological, surgical, combined, and alternative methods.^2^, ^3^ Identifying an optimal method is desirable as this is an important day‐to‐day practice clinical intervention.

Mechanical and pharmacological methods have distinct characteristics in terms of mechanism of action, effectiveness, and risks. Compared to pharmacological methods, balloon catheters have an additional benefit of a decreased risk of uterine hyperstimulation and a more favorable perinatal safety profile.^4^ When balloon catheters are compared to vaginal dinoprostone, both offer comparable effectiveness.^5^ However, balloon catheters show lower effectiveness when compared to oral (relative risk [RR] for cesarean delivery 1.17, 95% confidence interval [CI]: 1.04; 1.32) and vaginal misoprostol (RR for cesarean delivery, 1.28, 95% CI: 1.02; 1.60).^5^ Given these differences, the balance of safety and effectiveness has prompted the concept of cointervention, combined mechanical and pharmacological cervical ripening and IOL, which is gaining popularity due to the potential for faster delivery.^6^

Interventions that speed up labor need to be carefully assessed for safety aspects. A speed‐safety trade‐off may arise when trying to shorten the induction to delivery interval. Most randomized controlled trials (RCTs) using combined methods for IOL were powered for the time‐to‐delivery interval (quantitative variable) hence showing “positive” trial conclusions and were not powered to study important effectiveness (rate of vaginal birth) and safety outcomes.^7^, ^8^, ^9^, ^10^, ^11^, ^12^ Several RCTs^8^, ^11^, ^13^, ^14^, ^15^ and a few conventional meta‐analyses have been published on this topic with variable results.^16^, ^17^ However, all the aggregate data meta‐analyses showed multiple methodological flaws, including both oral and vaginal misoprostol preparations together, not excluding or stratifying according to high‐dose and low‐dose misoprostol preparations and including quasi‐experimental studies or retracted RCTs with trustworthiness concerns.^16^, ^17^, ^18^, ^19^, ^20^, ^21^ Hence, there is a research gap in the use of combined methods to inform clinical practice. To date, no major international guidelines have recommended the use of combined methods for cervical ripening in IOL, but the concurrent use of a balloon catheter and vaginal misoprostol is gaining popularity in clinical practice.^6^, ^12^

### 1.2 Objectives

We performed an individual participant data (IPD) meta‐analysis comparing the effectiveness and safety of cervical ripening in IOL using a balloon catheter with concurrent low‐dose vaginal misoprostol (combined group) with low‐dose vaginal misoprostol alone. Beyond standard meta‐analysis, we also explicitly focused on assessing the quality and trustworthiness of the evidence base.

## 2 METHODS

This international collaborative IPD meta‐analysis followed a protocol that was prospectively registered with the International Prospective Register of Systematic Reviews (PROSPERO CRD42023429368, on 24‐05‐2023) and a statistical analysis plan produced in advance of data lock and analysis (Appendix A). Findings are reported following the Preferred Reporting Items for Systematic Reviews and Meta‐Analysis (PRISMA)‐IPD Statement.^22^

### 2.1 Eligibility criteria, information sources, search strategy

RCTs comparing balloon catheter plus concurrent low‐dose vaginal misoprostol compared with low‐dose vaginal misoprostol alone for cervical ripening in IOL. As per the recent Cochrane review on “mechanical methods for induction of labor”,^1^ we only chose to include low‐dose misoprostol (defined as ≤50 mcg every ≥4 h) as evidence suggests that low‐dose misoprostol is superior to high‐dose misoprostol regarding safety outcomes and is equally effective. Cluster randomized trials, high‐dose misoprostol use, cross‐over and quasi‐experimental trials were excluded. Pregnant women with unfavorable cervices as defined by the modified Bishop score in each RCT and singleton fetuses at or beyond 34 weeks of gestation were included regardless of membrane status or previous cesarean delivery.

### 2.2 Study selection

Potentially eligible RCTs were identified from the 2019 Cochrane Review on “Mechanical Methods for Induction of Labor.”^1^ Databases included Ovid MEDLINE, Ovid Embase, Ovid Emcare, Scopus, Cochrane Pregnancy and Childbirth Group's Trials Register, the WHO International Clinical Trials Registry Platform (ICTRP), and clinicaltrials.gov were searched to identify further trials published since the Cochrane Review was conducted. The search strategy is outlined in Table S1. Reference lists of retrieved studies were searched further to identify eligible RCTs. The initial search was conducted in June 2023, with two search updates in January and July 2024. There were no language restrictions, and all the published and unpublished data were eligible. Two investigators (MP and FC) independently reviewed the identified titles and abstracts, followed by the full texts for eligibility, with disagreements solved by a third reviewer (WL).

### 2.3 Data access

We approached investigators of all potentially eligible RCTs to confirm eligibility further and requested those eligible to share individual participant‐level data. Contact details of the investigators were obtained through the published articles or the websites of their associated institutions, and invitations to participate were sent by weekly emails in the initial 2 months and spaced out afterwards, if there was no response. Where the primary or corresponding authors' contact details were unavailable, or no response was received, further attempts were made to contact other authors involved in the RCTs or through the contact details in their other publications. Lead/coauthors were copied in at least every month until July 2024. We used social media platforms and contacted institutions or clinics by email or phone when there was no response. Our academic contacts in particular countries were called to contact the authors/institutions who were not responding to the enquiries as the last attempt. When we had conducted multiple rounds of author enquiries using the above multimodality approaches over more than a year, the review team reached a consensus to declare the RCTs as “non‐shared” IPD. Once RCT investigators responded that they were interested in participating, regular emails were sent monthly, coordinating the data‐sharing process and clarifying details.

### 2.4 Data cleaning and evaluation for data integrity

RCT investigators supplied de‐identified participant‐level data, harmonized and recoded according to the pre‐defined IPD meta‐analysis codebook (Appendix B). Data were requested for all participants randomized, even if excluded from the original trial analyses. Where possible, the received data were examined for missing data, error, internal consistency, consistency with the publication, and pattern of treatment allocation and data presentation. Identified issues were communicated with RCT investigators for a solution before acceptance in the IPD meta‐analysis dataset.

### 2.5 Trustworthiness assessment of all the eligible RCTs

This was done using the trustworthiness in randomized controlled trials (TRACT) trustworthiness checklist (Appendix C).^23^ This screening tool aims to help identify and triage RCTs at risk of integrity issues. The checklist includes seven domains that apply to every RCT: governance, author group, plausibility of intervention usage, timeframe, drop‐out rates, baseline characteristics, and outcomes. Two review authors assessed all the RCTs published in full‐text form and disagreements were resolved by consensus. The categorization of “not meeting trustworthiness criteria” and “meeting trustworthiness criteria” was based on the overall TRACT checklist assessment across seven domains, a total score (cutoff of ≥10 considered for this comparison), year of publication, trial registration and availability of IPD for data checking.

### 2.6 Outcomes and effect modifiers

The primary outcomes (Table S2) included vaginal birth rate, a composite measure of adverse perinatal outcomes, and a composite measure of adverse maternal outcomes. The composite adverse perinatal outcome included at least one of stillbirth, neonatal death, neonatal Apgar score <7 at 5 min, acidosis (arterial umbilical cord pH <7.1), neonatal seizures, hypoxic–ischemic encephalopathy (HIE) grade, neonatal intensive care (NICU) admission, meconium aspiration syndrome, neonatal infection, cord prolapse, endotracheal intubation and/or external cardiac compressions. The composite adverse maternal outcome included admission to the intensive care unit, maternal infection, postpartum hemorrhage ≥1000 mL, maternal death, and uterine rupture. When calculating composite outcomes, the presence of one or more of these pre‐defined components of the composite outcome led to a positive composite maternal or perinatal outcome.

Secondary outcomes further explored the effectiveness and safety. Delivery outcomes included the mode of delivery, indications for an instrumental vaginal delivery or cesarean delivery, the cumulative rate of vaginal delivery, and total inpatient stay. Labor progression outcomes included uterine tachysystole, uterine hyperstimulation, oxytocin use, the presence of meconium‐stained liquor, and the use of a second method for cervical ripening. Individual secondary neonatal safety outcomes assessed included Apgar score <7 at 5 min, arterial umbilical cord pH <7.10, and NICU admission. Individual secondary maternal safety outcomes assessed included maternal infection, severe postpartum hemorrhage (≥1000 mL), and maternal intensive care unit admission. Potential effect modifiers of interest for the primary outcome vaginal birth included parity, maternal age, body mass index (BMI, calculated as weight in kilograms divided by the square of height in meters), initial Bishop score, and indications for labor induction (Table S2).

### 2.7 Assessment of risk of bias of included studies and certainty of evidence

The risk of bias (RoB) was independently assessed by two researchers (MP and FC) using the Cochrane RoB‐2 tool.^24^ Differences were resolved by consensus; if the information was insufficient, clarification was sought from trialists. We used the Grading of Recommendations Assessment, Development and Evaluation (GRADE) approach to assess the overall certainty of the evidence for the primary outcomes.^25^

### 2.8 Data synthesis

For each outcome, an intention‐to‐treat analysis was performed using all available data comparing the balloon catheter with concurrent low‐dose vaginal misoprostol to the use of low‐dose vaginal misoprostol alone for labor induction. The low‐dose vaginal misoprostol alone use was considered the reference group for all outcomes.

Our primary analysis strategy involved a two‐stage meta‐analysis method to synthesize the IPD. However, a one‐stage method was used as the primary analysis for outcomes with no events in any of the comparison groups of any included RCT. In the first step of the two‐stage method, we compared outcomes between the two groups for each included study, adjusting for maternal age and parity. For binary outcomes, we calculated odds ratios (ORs) along with 95% confidence intervals (CIs) using logistic regression. For the cumulative rate of vaginal births, which is a time‐to‐event outcome, we estimated subdistribution hazard ratios (sHRs) and 95% CIs using the subdistribution hazard model, which considered cesarean birth as a competing risk. In the second step, we combined the generated relative estimates using random‐effects models (restricted maximum likelihood estimator with Hartung‐Knapp‐Sidik‐Jonkman variance correction).^26^, ^27^ For the one‐stage method, we used multilevel mixed‐effects logistic regression (a stratified intercept by study and a random treatment effect, covariates as fixed effects, and maximum likelihood estimator) adjusting for maternal age and parity.^27^ We tested treatment‐covariate interactions for vaginal birth using interaction terms between treatment and potential effect modifiers, also adjusting for maternal age and parity. To avoid ecologic bias, we only considered within‐trial interaction. All variables besides the identification variable were checked for missing values and entries outside the expected ranges. In the event of missing values for covariates or potential effect modifiers in any RCT, we performed multiple imputations using chained Equations (10 imputed datasets) within the RCT before the meta‐analysis.

We extracted aggregate data for vaginal birth from all eligible RCTs, including those that did not contribute IPD. We performed an aggregate data meta‐analysis using the same random‐effects model to understand the difference in effect estimates between shared and non‐shared RCTs, and between those with and without trustworthiness concerns. A funnel plot was created to assess for evidence of publication bias. A one‐stage sensitivity analysis was planned for the primary outcomes and an as‐treated analysis to compare with the intention‐to‐treat analysis.

Statistical analyses were performed using the R statistical environment (Foundation for Statistical Computing, Vienna, Austria).^28^ The meta package was used for two‐stage meta‐analysis and aggregate data meta‐analysis,^29^ the lme4 package was used for a one‐stage meta‐analysis,^30^ and the cmprsk package was used for competing risk analysis.^31^

### 2.9 Role of the funding source

These funding sources had no role in the design, execution, analyses, or data interpretation.

## 3 RESULTS

### 3.1 Descriptive statistics of the search results

#### 3.1.1 Study selection and participants

**FIGURE 1:**
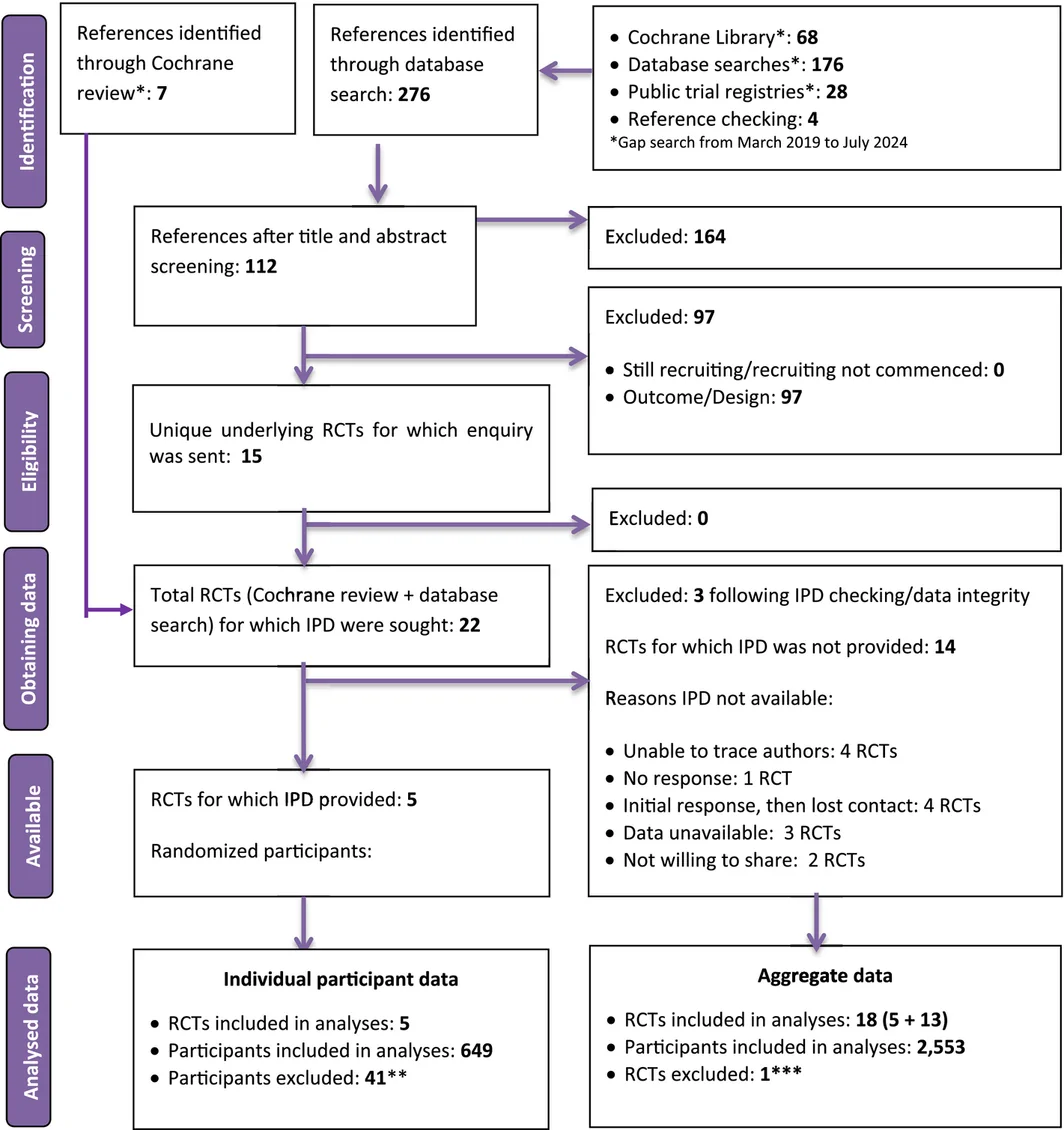
Identification of randomized controlled trials on methods of induction of labor evaluating the use of cervical balloon plus low‐dose misoprostol versus low‐dose misoprostol alone (PRISMA‐IPD flow diagram). RCTs, randomized controlled trials. *de Vaan MD, et al. Mechanical methods for induction of labor. *Cochrane Database Syst Rev*. 2019 Oct 18;10(10):CD001233 (from inception to March 2019). **23 pregnancies with intrauterine fetal death and 18 pregnancies with less than 34 weeks were excluded from Osoti 2018 et al. ***Aggregate could not be extracted for the desired comparison from Polonia‐Valente 2023 et al.

Our systematic search identified 276 unique references, and seven were added from the 2019 Cochrane review on “mechanical methods for induction of labor” (Figure 1).^1^ After screening, 22 RCTs were potentially eligible for inclusion, and enquiries were sent. A total of 14 of the 22 RCTs (63.6%) did not participate in this IPD meta‐analysis. These 14 RCTs did not share data for the following reasons: two author groups declined to share the data, raw data were unavailable from three RCTs, four author groups initially responded but then discontinued contact, four author groups could not be traced, and there was no response from one group. Table 2 shows the characteristics of RCTs that did not share IPD. Overall, of the 22 RCTs, 59.1%, 13 (3 shared and 10 non‐shared) RCTs were identified as “not meeting trustworthiness criteria.”

#### 3.1.2 Study characteristics and trustworthiness assessment of the shared RCTs

Of 22 RCTs, eight shared data for this IPD meta‐analysis. Following IPD checking, three RCTs (604 participants) had to be excluded due to data integrity and trustworthiness concerns. The RCT by Kalambe et al.^32^ was excluded as the dataset had multiple rows of data duplications and large sequences of patients with the exactly same induction‐to‐delivery interval and other variables. Also, none of the reported neonatal or maternal outcomes were in the dataset.^32^ The RCT by Viteri et al.^14^ had evidence of multiple rows of data duplications for all the variables. The RCT by Kadu et al.^15^ was excluded as the data did not show evidence of randomization, and multiple pairs of identical data were noticed. We sought an explanation via emails from the relevant author groups, and none of these discrepancies were explained by the authors.

We included the remaining five eligible RCTs that shared IPD (649 participants) in this meta‐analysis.^7^, ^8^, ^13^, ^33^, ^34^ All the included RCTs were recent (2014–2021). Although all RCTs were published within the last 10 years, only two RCTs had prospective trial registration.^8^, ^34^ Two had retrospective registrations,^7^, ^13^ and another was unregistered.^33^ However, after IPD checking, there was no reason to exclude these three RCTs with retrospective and absent registrations. Table 1 summarizes the study and baseline characteristics of the five participating RCTs.

**TABLE 1:** Characteristics of randomized controlled trials that provided eligible individual participant data.

Author, year and reference	Published, conference abstract or registered only	Country	Trial registration (prospective, retrospective or unregistered)	Combined (balloon catheter plus low‐dose vaginal misoprostol)	Low‐dose vaginal misoprostol alone	Inclusion criteria	No. of participants	Maternal age in years, mean (SD)	BMI, mean (SD)	Gestational age in weeks, median (IQR)	Initial Bishop score^a^, median (IQR)	Nulliparous *n*/*N* (%)	Multiparous *n*/*N* (%)
								Combined group versus low‐dose vaginal misoprostol alone group					
Lanka et al. (2014)^7^, ^a^	Published	India	Retrospective	A 16‐Fr Foley catheter with 30 mL of sterile water and traction applied + misoprostol 25 mcg vaginal tablet at the same time. The Foley catheter was retained in situ until spontaneous expulsion or for a maximum of 12 h. A 25‐mcg misoprostol was repeated 4‐hourly, up to a maximum of 8 doses if the BS was ≤6	25 mcg misoprostol tablet was inserted vaginally into the posterior fornix and the cervix was assessed every 4 h. A 25 mcg misoprostol was inserted 4‐hourly, up to a maximum of 8 doses if the BS was ≤6	Primip + multip Low‐risk women Gest: ≥ 28 ROM: No BS ≤4 Prev CS: No	126 (63 vs. 63)	26.4 (3.7) vs. 25.9 (3.3)	25.6 (3.9) vs. 25.6 (3.7)	39.0 (2.0) vs. 40.0 (3.0)	2.0 (1.0) vs. 2.0 (0.98)	50/63 (79.4) vs. 50/63 (79.4)	13/63 (20.6) vs. 13/63 (20.6)
Al‐Ibraheemi et al. (2018)^8^, ^d^	Published	USA	Prospective	A Foley catheter with 60 mL of normal saline and traction applied + misoprostol 25 mcg vaginal tablet at the same time. The Foley catheter was retained in situ until spontaneous expulsion or for a maximum of 24 h. A 25‐mcg misoprostol inserted vaginally into the posterior fornix every 4 h and repeated up to a maximum of 6 doses	25 mcg misoprostol tablet inserted vaginally into the posterior fornix every 4 h and repeated up to a maximum of 6 doses	Primip + multip Low‐risk women Gest: ≥37 ROM: No BS ≤6 Prev CS: No	200 (100 vs. 100)	31.8 (6.4) vs. 31.5 (6.0)	31.4 (5.8) vs. 31.6 (6.9)	40.0 (2.2) vs. 39.3 (2.8)	3.0 (2.0) vs. 2.0 (3.0)	70/100 (70) vs. 74/100 (74)	30/100 (30) vs. 26/100 (26)
Gilani et al. (2018)^33^	Published	Pakistan	Unregistered	A 16‐Fr Foley catheter with 60 mL of normal saline and traction applied + misoprostol 50 mcg vaginal tablet at the same time. The Foley catheter was retained in situ until spontaneous expulsion or for a maximum of 12 h. A 50 mcg misoprostol tablet inserted vaginally into the posterior fornix was repeated at 6 and 12 h up to a maximum of 3 doses	50 mcg misoprostol tablet inserted vaginally into the posterior fornix was repeated at 6 h and 12 h up to a maximum of 3 doses	Primip + multip Low‐risk women Gest: ≥ 38 ROM: No BS ≤ NS Prev CS: No	96 (48 vs. 48)	28.1 (4.1) vs. 27.8 (4.3)	Not reported	40.0 (1.0) vs. 40.0 (2.0)	Not reported	6/48 (12.5) vs. 6/48 (12.5)	42/48 (87.5) vs. 42/48 (87.5)
Osoti et al. (2018)^13^	Published	Kenya	Retrospective	An 18‐Fr Foley catheter with 30 mL of sterile water and traction applied + misoprostol 25 mcg vaginal tablet at the same time. A 25‐mcg misoprostol inserted into the posterior fornix 6 hourly up to a maximum of 6 doses	25 mcg of misoprostol inserted into the posterior fornix 6 hourly up to a maximum of 4 doses	Primip + multip Low‐risk women Gest: ≥28 ROM: No BS ≤6 Prev CS: No	180^b^ (63 vs. 76)	28.4 (6.0) vs. 26.5 (4.6)	Not reported	40.0 (2.0) vs. 41.0 (1.8)	Not reported	39/63 (61.9) vs. 38/76 (50.0)	24/63 (38.1) vs. 38/76 (50.0)
Romero Ramirez et al. (2021)^34^	Registered only	Spain	Prospective	A 16‐Fr Foley catheter with 30 mL of normal saline and traction applied + misoprostol 25 mcg vaginal tablet at the same time. The Foley catheter was retained in situ until spontaneous expulsion or for a maximum of 12 h. A 25 mcg of misoprostol inserted into the posterior fornix 4 hourly up to a maximum of 5 doses	25 mcg of misoprostol inserted into the posterior fornix 4 hourly up to a maximum of 5 doses	Primip + multip Low‐risk women Gest: ≥37 ROM: No BS ≤6 Prev CS: No	88^c^ (49 vs. 39)	31.6 (5.5) vs. 31.3 (5.2)	27.9 (6.6) vs. 24.8 (4.9)	40.6 (2.6) vs. 41.0 (1.9)	2.0 (3.0) vs. 2.0 (2.0)	23/49 (46.9) vs. 24/39 (61.5)	26/49 (53.1) vs. 15/39 (38.5)

*Note*: BMI, calculated as weight in kilograms divided by the square of height in meters.

Abbreviations: BMI, body mass index; BS, Bishop score; CS, cesarean section; IQR, interquartile range; multip, multiparous women; NS, not stated; Primip, primiparous women; ROM, rupture of membranes; SD, standard deviation.

^a^ All the 126 women were more than 35 or more weeks of gestation.

^b^ 23 pregnancies with intrauterine fetal death and 16 pregnancies with less than 34 weeks were excluded from this meta‐analysis.

^c^ The dataset was from an unpublished trial that was halted due to poor recruitment.

^d^ Maternal race/ethnicity was reported only by Al‐Ibraheemi et al.^8^

#### 3.1.3 Study characteristics and trustworthiness assessment of non‐shared RCTs

Characteristics of RCTs for which enquiry was sent but did not contribute to the IPD meta‐analysis are shown in Table 2 (*n* = 14). Of these 14 non‐shared RCTs, 10 (71.4%) were identified as “not meeting trustworthiness criteria.” Further, Table S3 shows the distribution of trustworthiness ratings across seven domains of the TRACT checklist among non‐shared RCTs identified as “not meeting trustworthiness criteria.” Although recent (2017–2022), none had prospective registration. Multiple concerns were noticed over all domains except for the domains on the author group and the timeframe of the studies.

**TABLE 2:** Characteristics of randomized controlled trials for which enquiry was sent but did not contribute to IPD meta‐analysis (*n* = 14).

First author, year and reference	Full paper published or conference abstract only	Country	Trial registration (prospective, retrospective or unregistered)	*N*	Study population	Reason for not contributing IPD^a^	Trustworthy assessment outcome (TRACT checklist^23^ and eyeballing the paper)
Carbone et al. (2013)^11^	Full paper	USA	Prospective	123	Women undergoing induction of labor with singleton pregnancies at 24 weeks of gestation or greater with an unfavorable cervix (Bishop score 6 or lower)	Data no longer available (data destroyed)	‘Meeting trustworthiness criteria’
Ugwu et al. (2013)^10^	Full paper	Nigeria	Unregistered	150	Women with singleton gestation at term (≥ 37 weeks gestation) with the cephalic presentation, intact fetal membranes, unfavorable. Bishop score (≤5) and a normal fetal heart rate	Data no longer available (data destroyed)	“Meeting trustworthiness criteria”
Aduloju, 2016^42^	Full paper	Nigeria	Prospective	210	Women with live singleton fetuses with cephalic presentation, intact membranes, reactive non‐stress test and unfavorable cervices (Bishop score <6) scheduled to undergo IOL at term	Data no longer available (data destroyed)	“Meeting trustworthiness criteria”
Bhatiyani et al. (2017)^19^	Full paper	India	Unregistered	105	Women with a singleton, viable gestation 28 weeks or greater, cephalic presentation, intact membranes and an unfavorable cervix (Bishop score 6 or less)	Not willing to share	“Not meeting trustworthiness criteria”
Ashwini, 2018^43^	Full paper	India	Unregistered	100	Singleton pregnancies, vertex presentation, unfavorable cervix (Bishop score <6), gestational age greater than 28 weeks' gestation	Not willing to share	“Not meeting trustworthiness criteria”
El‐Kelani, 2019^44^	Full paper	Egypt	Unregistered	200	Women undergoing induction of labor with singleton pregnancies at full‐term gestation with an unfavorable cervix (Bishop score ≤6)	No response after initial positive response	“Not meeting trustworthiness criteria”
Kareem, 2020^45^	Full paper	Iraq	Unregistered	120	Women were primigravida with gestational age ≥37 weeks singleton viable fetus, cephalic presentation with intact membranes, absence of labor and a modified Bishop score of <4	No response after initial positive response	“Not meeting trustworthiness criteria”
Aregeb, 2021^46^	Full paper	Egypt	Unregistered	72	Women with gestational age (≥37 weeks), vertex presentation with intact fetal membranes, normal fetal non‐stress test, modified Bishop score ≤5 and cervical dilation less than or equal to 2 cm	Unable to trace authors	“Not meeting trustworthiness criteria”
Devi, 2021^47^	Full paper	India	Unregistered	200	Women with term singleton, live pregnancy, cephalic presentation, intact membranes and unfavorable cervix (Bishop score <6)	Unable to trace authors	“Not meeting trustworthiness criteria”
Izevbigie, 2021^48^	Full paper	Nigeria	Unregistered	172	Women admitted for induction of labor with no contraindication for vaginal delivery, reactive preinduction cardiotocograph, singleton fetus in longitudinal lie, cephalic presentation, gestational age between 37 weeks and below 41 weeks and 6 days, Bishop score of less than 6 and an estimated fetal weight of ≥2.5 kg to ≤4 kg	Unable to trace authors	“Not meeting trustworthiness criteria”
Sharma, 2021^50^	Full paper	India	Unregistered	242	Women with 34 to 41 weeks period of gestation, singleton live fetus, cephalic presentation, modified Bishop score ≤6 and intact membranes	Unable to trace authors	“Not meeting trustworthiness criteria”
Elpo, 2023^51^	Full paper	Brazil	Retrospective	230	Women with normal‐risk pregnancies, aged between 18 and 40 years, admitted for late‐term labor induction, and with a Bishop index ≤6 at admission	No response	“Not meeting trustworthiness criteria”
Toshniwal, 2022^49^	Full paper	India	Unregistered	110	Women with singleton gestation, vertex presentation, gestation >39 weeks, Bishop score <6, no contraindication for normal vaginal delivery, post‐term pregnancy and low‐risk pregnancy	No response after initial positive response	“Not meeting trustworthiness criteria”
Polonia‐Valente et al. (2023)^9^	Full paper	Portugal	Retrospective	2023	Women with ≥37 weeks of gestation, singleton, vertex presenting gestations, no contraindication to vaginal delivery, intact membranes, Bishop score <7 and cervical dilation ≤2 cm	No response after initial positive response	“Meeting trustworthiness criteria”

Abbreviations: AFI, amniotic fluid index; BS, Bishop score; CS, cesarean section; IOL, induction of labor; IPD, individual participant data; multip, multiparous women; nullip, nulliparous women; NS, not stated; ROM, rupture of membranes.

^a^ Regular multimodality author enquiry was carried out over 1 year.

#### 3.1.4 Risk of bias of included studies

**FIGURE 2:**
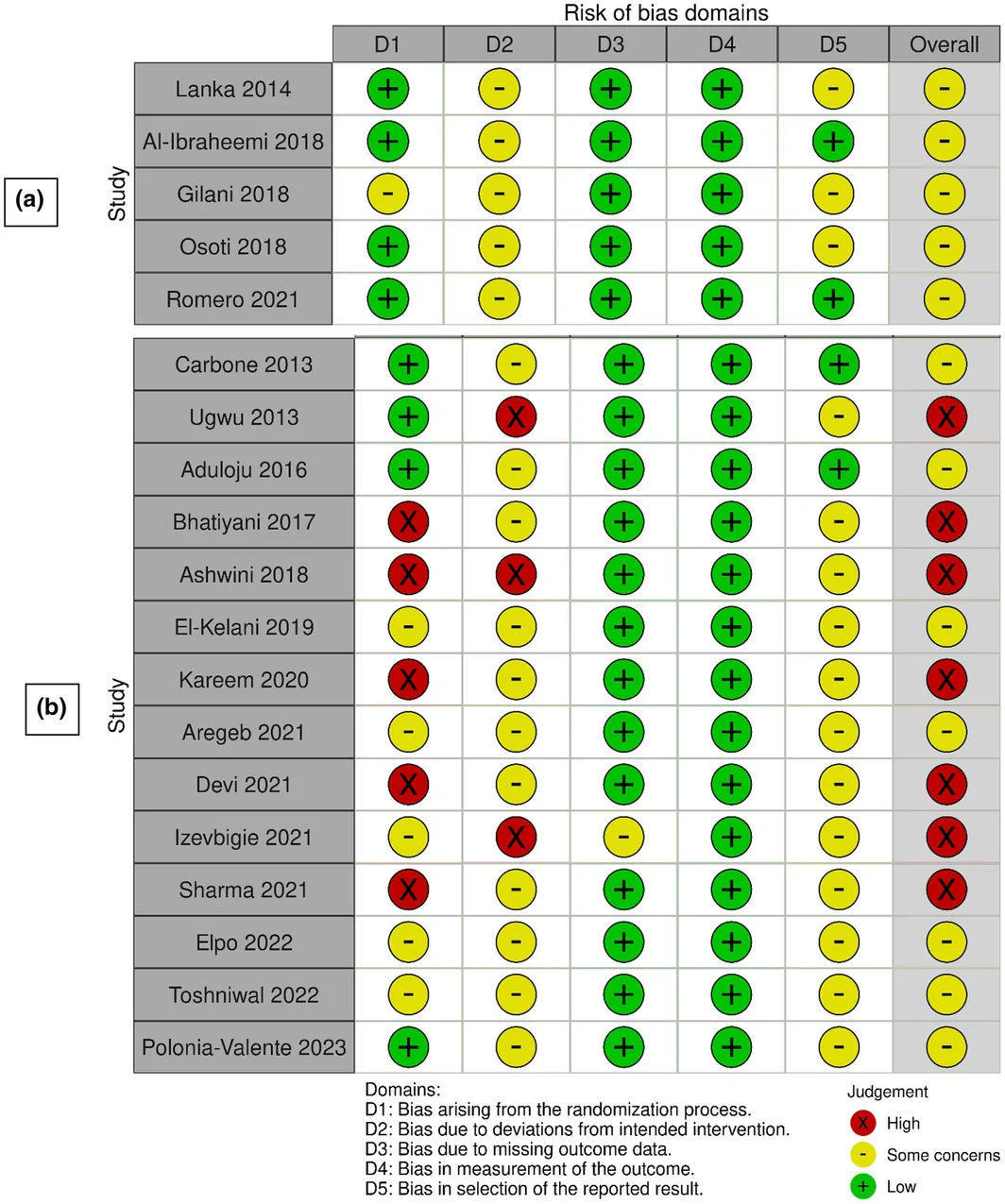
Risk of bias assessment of participating and non‐participating randomized controlled trials. (a) Risk of bias assessment of IPD shared (‘participating’) randomized controlled trials. (b) Risk of bias assessment of IPD non‐shared (‘not participating’) randomized controlled trials. IPD: Individual participant data.

On screening for risk of bias, RCTs were mostly identified as having overall “some concerns” largely owing to the inability to blind the participants and/or investigator due to the nature of the intervention (Figure 2 and Figure S1). Figure 2 and Figure S1 also show the risk of bias summary for 14 RCTs that did not provide IPD, with seven RCTs showing overall “high risk.”

### 3.2 Results of IPD meta‐analysis

Of the 649 included participants, 323 (49.8%) participants were allocated to the combined group, and 326 (50.2%) were allocated to the vaginal misoprostol alone group. There were 380 (58.6%) nulliparous and 269 (41.4%) multiparous women.

#### 3.2.1 Primary outcomes

**FIGURE 3:**
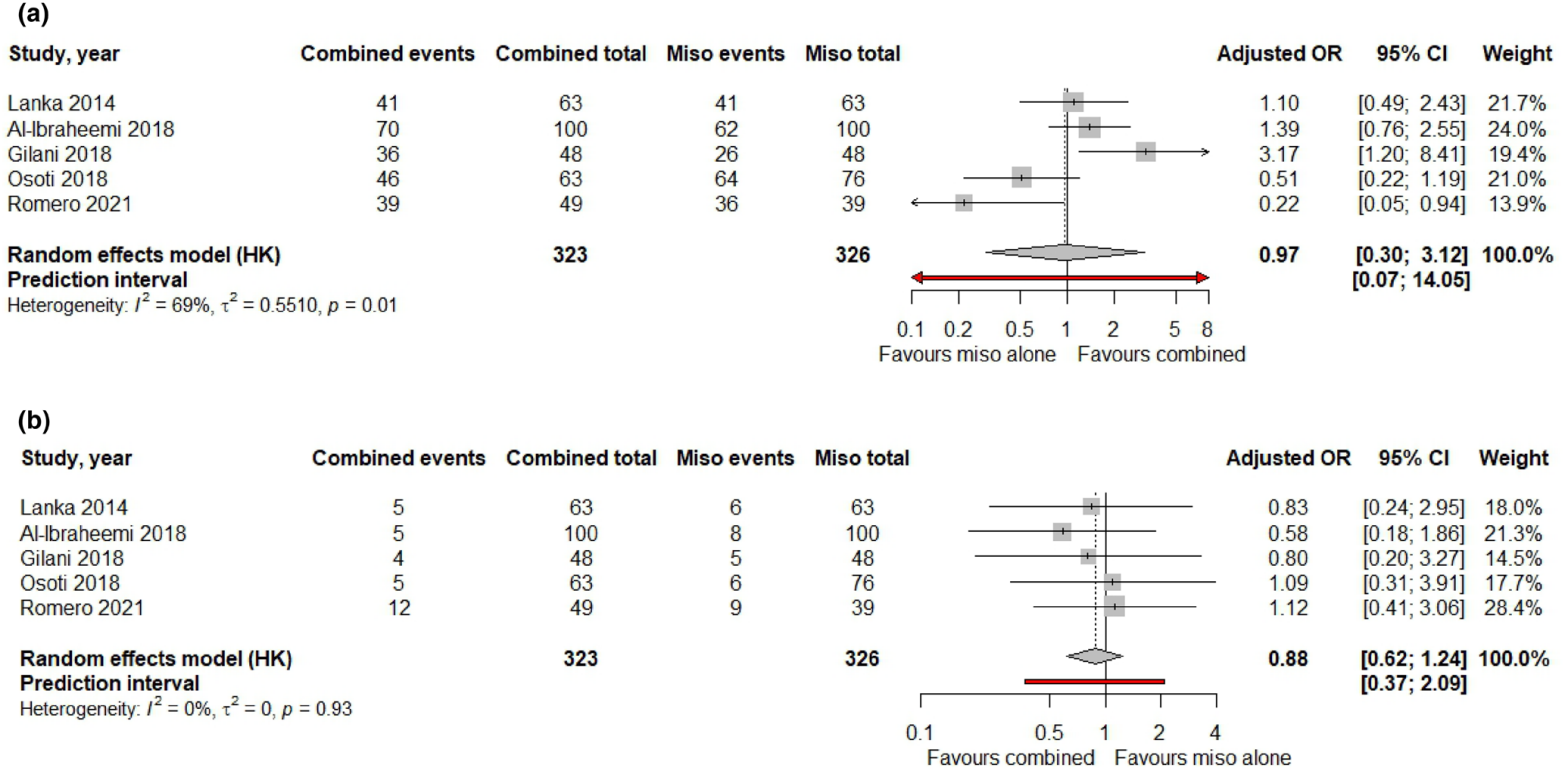
Forest plots of individual participant data (IPD) meta‐analyses of the primary outcomes comparing the use of cervical balloon plus low‐dose misoprostol (combined) versus low‐dose misoprostol alone. (a) Primary outcomes: Odds of vaginal delivery between two methods (two‐stage). (b) Primary outcomes: Composite of adverse perinatal outcomes between two methods (two‐stage). 95% CI, 95% confidence interval; HK, Hartung‐Knapp‐Sidik‐Jonkman variance correction. Composite maternal outcomes were analyzed with the one‐stage method.

The odds of vaginal delivery were comparable between combined and low‐dose vaginal misoprostol alone groups (five RCTs, 649 women, 71.8% vs. 70.2%, aOR 0.97, 95% CI: 0.30; 3.12, *P* = 0.94, *I* ^2^ = 69%, very low certainty of evidence as downgraded due to imprecision, inconsistency and data completeness, Figure 3a). The odds of composite adverse perinatal outcomes (5 RCTs, 649 women, 9.6% vs. 10.4%, aOR 0.88, 95% CI: 0.62; 1.24, *P* = 0.35, *I* ^2^ = 0%, very low certainty of evidence as downgraded due to imprecision, inconsistency and data completeness, Figure 3b) were comparable between two groups. The odds of composite adverse maternal outcomes (5 RCTs, 465 women, 5.7% vs. 7.5%, aOR 0.70, 95% CI: 0.33; 1.50, *P* = 0.36, very low certainty of evidence as downgraded due to imprecision, inconsistency and data completeness) were also comparable between two groups.

#### 3.2.2 Secondary outcomes

Table 3 and Figures S2–S12 show the secondary outcomes for the combined method versus low‐dose vaginal misoprostol alone for cervical ripening before IOL. Time‐to‐event analysis for the hazard ratio of vaginal birth (Table 3 and Figure S6) did not show a difference and demonstrated substantial heterogeneity (*I* ^2^ = 73%). Women in the combined group reported significantly less uterine hyperstimulation (5 RCTs, 649 women, 3.4% vs. 12.6%, aOR 0.20, 95% CI: 0.10; 0.41, *P* < 0.001, Table 3) and less meconium‐stained liquor (3 RCTs, 414 women, 4.7% vs. 17.3%, aOR 0.23, 95% CI: 0.07; 0.77, *P* = 0.03, *I* ^2^ = 0%, Table 3 and Figure S8). None of the other individual perinatal or maternal outcomes significantly differed between the two methods (Table 3 and Figures S11 and S12).

**TABLE 3:** Secondary outcomes of balloon catheter plus concurrent low‐dose vaginal misoprostol (combined) versus low‐dose vaginal misoprostol alone for cervical ripening induction of labor.

Secondary outcome	No. of trials	No. of women	Crude incidence (%)—combined versus low‐dose vaginal misoprostol alone	aOR (95% CI)	*I* ^2^ (95% CI)	Analysis method^a^
Delivery outcomes						
Cesarean delivery	5	649	28.2 vs. 29.7	1.03 (0.32; 3.32)	69 (21; 88)	Two‐stage
Cesarean delivery for failure to progress	4	510	12.7 vs. 18.0	0.67 (0.19; 2.30)	41 (0; 80)	Two‐stage
Cesarean delivery for fetal distress	4	510	13.8 vs. 13.2	1.12 (0.66; 1.88)	—	One‐stage
Unassisted vaginal birth	4	553	59.3 vs. 59.7	0.91 (0.42; 196)	39 (0; 79)	Two‐stage
Instrumental vaginal birth	3	414	15.6 vs. 18.3	0.85 (0.26; 2.81)	2 (0; 90)	Two‐stage
Instrumental vaginal birth for failure to progress	2	288	5.4 vs. 4.3	1.30 (0.39; 4.29)	—	One‐stage
Instrumental vaginal birth for fetal distress	2	288	7.4 vs. 13.7	0.27 (0.03; 2.76)	—	One‐stage
Time to vaginal birth (hours)	5	649	16.0 vs. 19.0^b^	sHR 1.41 (0.83; 2.40)	73 (33; 89)	Two‐stage
Labor progression outcomes						
Uterine tachysystole	—	—	Insufficient data	—	—	—
Uterine hyperstimulation	5	649	3.4 vs. 12.6	0.20 (0.10; 0.41)	—	One‐stage
Oxytocin use	3	414	59.9 vs. 56.4	1.47 (0.23; 9.58)	69 (0; 91)	Two‐stage
Meconium‐stained liquor	3	414	4.7 vs. 17.3	0.23 (0.07; 0.77)	0 (0; 90)	Two‐stage
Use of epidural analgesia	3	414	88.2 vs. 88.1	1.00 (0.20; 4.93)	16 (0; 91)	Two‐stage
Use of more methods for cervical ripening (second methods)	—	—	Insufficient data	—	—	—
Maternal safety outcomes						
Maternal infection	2	326	4.3 vs. 7.4	0.58 (0.04; 8.13)	—	Two‐stage
Severe postpartum hemorrhage (≥1000 mL)	—	—	Insufficient data	—	—	—
Maternal ICU admission	—	—	Insufficient data	—	—	—
Perinatal safety outcomes						
Cord prolapse	—	—	Insufficient data	—	—	—
Apgar score <7 at 5 min 126	3	414	0.9 vs. 3.0	0.33 (0.06; 1.65)	—	One‐stage
Arterial umbilical cord pH <7.10	2	288	9.4 vs. 10.1	0.91 (0.76; 1.10)	—	Two‐stage
NICU admission	5	649	5.9 vs. 6.1	1.00 (0.48; 2.09)	0 (0; 79)	Two‐stage

Abbreviations: aOR, adjusted odds ratio; ICU, intensive care unit; NICU, neonatal intensive care unit; RR, rate ratio; sHR, subdistribution hazard ratio; SMD, standardized mean difference.

^a^ Two‐stage as the primary strategy, one‐stage used when zero events are encountered in any arm of any included study.

^b^ Median values have been reported.

With regard to the intervention‐covariate interactions, no differential impact of treatment on the odds of vaginal delivery (Table S4) was identified for baseline characteristics as potential effect modifiers. Sensitivity analyses using the one‐stage method and “as‐treated” analysis for primary outcomes were not performed due to the limited sample size and uncertain effect estimates.

### 3.3 Results of aggregate data meta‐analysis and differential results based on the trustworthiness concerns

**FIGURE 4:**
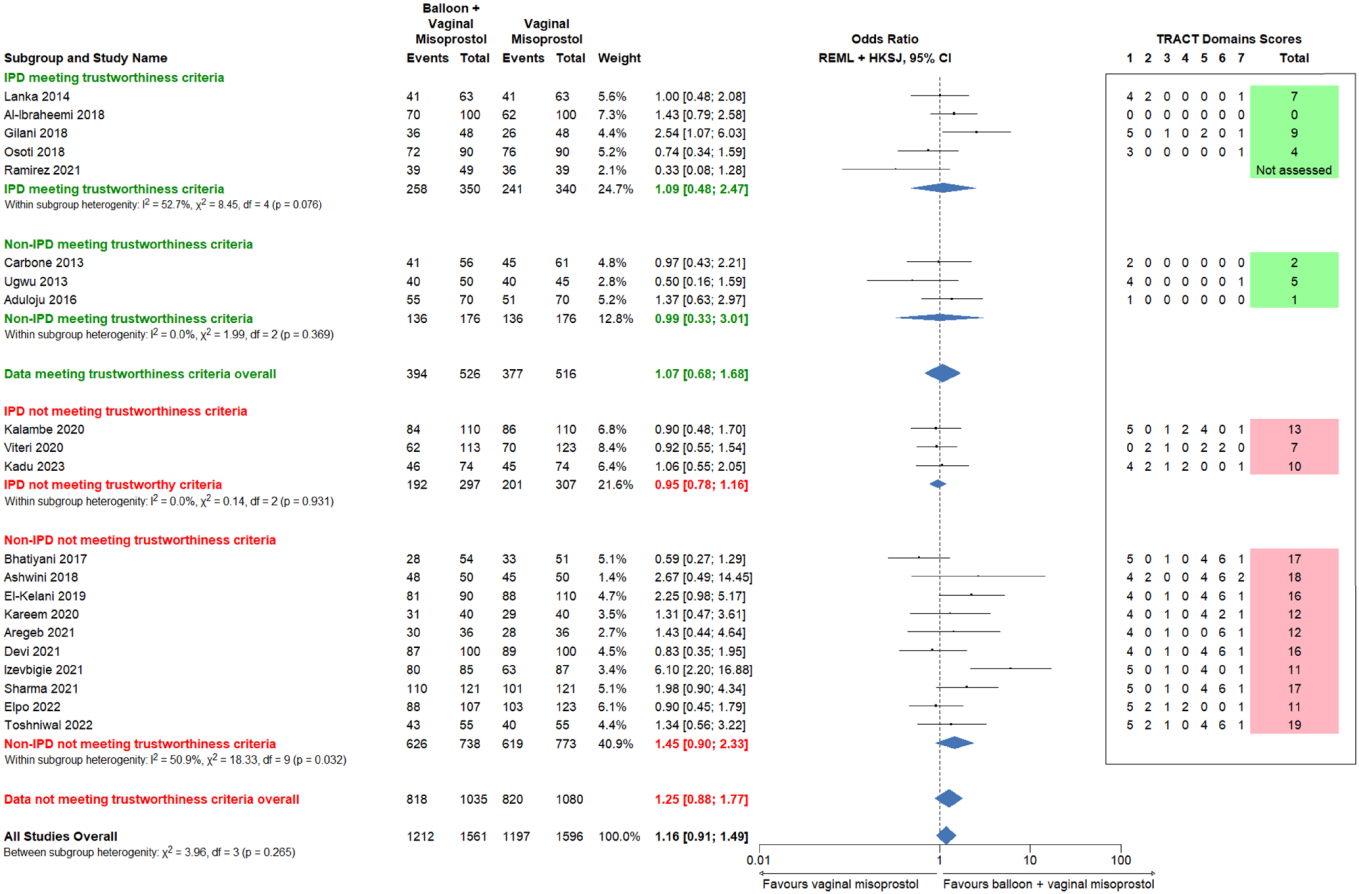
Aggregate data meta‐analysis for vaginal delivery based on the trustworthiness assessment outcomes for the randomized controlled trials comparing the use of cervical balloon plus low‐dose misoprostol (combined) versus low‐dose misoprostol alone. REML + HK: Random Effects Model with Hartung‐Knapp‐Sidik‐Jonkman variance correction; 95% CI: 95% confidence interval; For Romero 2021 et al, TRACT^1^ assessment was not done as it is unpublished. However, it met our trustworthiness criteria with other modalities of integrity checks, as described in the paper. No summary data were found for one non‐shared RCT by Polonia‐Valante et al, 2023. Mol BW, Lai S, Rahim A, Bordewijk EM, Wang R, van ER, et al. Checklist to assess Trustworthiness in RAndomised Controlled Trials (TRACT checklist): concept proposal and pilot. *Res Integr Peer Rev*. 2023;8(1):6.

We could extract summary data for vaginal birth from 13 non‐IPD sharing RCTs and eight IPD RCTs (Figure 4). No summary data were found for one non‐shared RCT by Polonia‐Valente et al.^9^ Figure 4 summarizes the variations of the meta‐analysis results with the trustworthiness concerns. The pooled odds ratio for vaginal delivery with aggregate data from eight RCTs “meeting trustworthiness criteria” (IPD and non‐IPD) was 1.07 (95% CI: 0.68; 1.68) and from 13 RCTs “not meeting trustworthiness criteria” (IPD and non‐IPD) was 1.25 (95% CI: 0.88; 1.77) (Figure 4). The funnel plot of all RCTs (participating and non‐participating) shows that all RCTs fall around the middle, representing a low risk of publication bias (Figure S13).

## 4 DISCUSSION

Based on trustworthy data, the current evidence does not suggest that one method is superior to the other for the effectiveness of the combined balloon catheter with concurrent low‐dose vaginal misoprostol versus low‐dose vaginal misoprostol alone. The safety of balloon catheter with concurrent low‐dose vaginal misoprostol for cervical ripening in IOL is still uncertain due to the low data retrieval rate and common trustworthiness concerns among the underlying RCTs on this topic. The results could slightly favor the combined method due to reduced hyperstimulation and meconium‐stained liquor.

### 4.1 Comparison with existing literature

A recent conventional meta‐analysis by Yin et al.^16^ summarizing the outcomes of 13 RCTs has shown that a combined method for cervical ripening was not associated with significant reductions in time‐to‐vaginal delivery. There have been significant reductions in uterine tachysystole and NICU admissions. However, this meta‐analysis has included RCTs with both low‐ and high‐dose misoprostol. Nasioudis et al.^17^ showed that a combination of a mechanical method with misoprostol for cervical ripening was associated with a shorter time‐to‐delivery, a comparable cesarean delivery rate, and a lower incidence of NICU admissions compared with the use of misoprostol alone. This meta‐analysis has pooled both oral and vaginal misoprostol with low‐dose and high‐dose preparations together. Given that our study does not suggest one method is superior to the other, the dose and the route of administration of misoprostol for the combined method could impact the effectiveness and safety outcomes. The FIGO has recommended the use of either oral or vaginal misoprostol for cervical ripening in 2023.^35^ The recent Finnish RCT comparing balloon catheter, oral misoprostol, or a combination of both for cervical ripening in late‐term and post‐term nulliparous women has shown a shorter induction to delivery interval and comparable rates of cesarean section and neonatal outcomes compared to either method alone.^36^ This implies the potential benefit of using the combined method.

A meta‐analysis by Ornat et al.^18^ has concluded that the combined use of misoprostol and a cervical balloon catheter reduces the induction‐to‐delivery interval and the number of NICU admissions in women induced with an unfavorable cervix. However, this meta‐analysis has included high‐dose regimes, RCTs with trustworthiness concerns,^19^ and a retracted RCT.^20^ A recent meta‐review summarizing 11 systematic reviews, including most of the above, resulting in 207 randomized and quasi‐randomized trials, has concluded that the combination of a single‐balloon catheter with misoprostol was the most effective method in reducing the odds of cesarean delivery and prolonged time‐to‐vaginal delivery.^21^ NICU admissions were also less common with the combined method. This meta‐review differs from the other independent pairwise comparisons mentioned earlier by being a network meta‐analysis and only included RCTs already included in meta‐analyses, missing newer trials. Moving forward, our IPD meta‐analysis, along with its detailed analysis and inclusion of trustworthy RCTs, becomes an important addition to the evidence synthesis of this comparison.

A recent commentary by Wang et al. has discussed about a meta‐analysis by Katsika et al. on an assisted reproduction topic where no RCTs are left following integrity checks.^37^, ^38^ Before this study, these 13 RCTs contributed to 11 meta‐analyses without a trustworthiness assessment and showed positive results. The same scenario has happened in the present meta‐analysis.

### 4.2 Strengths and limitations

Unlike previous aggregate‐data meta‐analyses on the topic, in the current IPD meta‐analysis we undertook a formal assessment of the trustworthiness of individual RCTs and evaluated how trustworthiness affects the effect estimates. This concept has been further evaluated in a meta‐epidemiological study by the authors.^39^ As highlighted by the OBGYN Editors' Integrity Group (OGEIG) in the recently published trustworthiness criteria for meta‐analyses of RCTs, assessing the trustworthiness of RCTs is crucial to the integrity of meta‐analyses.^40^, ^41^ When it comes to clinical practice, clinicians need optimal methods for cervical ripening in IOL in terms of both effectiveness and safety. Speed‐safety trade‐off should not jeopardize this approach when compared to safer single‐agent preparations. Less uterine hyperstimulation and less meconium‐stained liquor in the combined group might be related to the fewer doses of misoprostol doses, which demonstrated the better‐balancing safety effects of the cointervention methods. Given no apparent increase in the effectiveness and uncertainty of the safety outcomes of this intervention, balloon catheter with concurrent vaginal misoprostol cannot be justified for routine clinical practice and inclusion in the practice guidelines in the present stage. However, the results could slightly favor the combined method, given reduced hyperstimulation and meconium‐stained liquor with the combined method. This information should be used when making decisions for cervical ripening with shared decision making.

The main strength of the present study is the integrated trustworthiness assessment for all eligible RCTs to assess the quality and trustworthiness of the evidence base. The trustworthiness assessment has now been recognized as a crucial component of the integrity of meta‐analyses. By integrating the trustworthiness assessment of RCTs using the TRACT checklist, the reliability of this work has been enhanced. Additionally, the pre‐defined analysis plan, the use of a thoroughly cleaned IPD database, and the presentation of both IPD and aggregate data meta‐analyses are other strengths of this work.

Our IPD meta‐analysis sample size was hampered by the exclusion of three shared RCTs, which decreased the sample size and the statistical power of the study. The authors acknowledge the loss of IPD from 14 non‐participating RCTs as a limitation despite active author enquiries to obtain the data. On the other hand, only four of these 14 RCTs “met trustworthiness criteria.” Therefore, one should not see the unavailability of data from these RCTs not meeting trustworthiness criteria as a limitation but rather a strength of this work, given those are unreliable data. Moreover, we have also performed an aggregate data meta‐analysis to show the differential effect from trustworthy or untrustworthy data, which showed more extreme pooled estimates favoring the intervention with untrustworthy data even though this was not statistically significant. This further increases the value of this study. All the non‐shared RCTs were published after 2017, and none had prospective trial registration. Due to the limited sample size, we could not demonstrate clear differences in effectiveness and safety outcomes, leading to very low certainty of evidence.

### 4.3 Conclusions and implications

Most RCTs comparing balloon catheter with concurrent vaginal misoprostol versus vaginal misoprostol alone for cervical ripening are of low quality and have trustworthiness concerns. Trustworthy data suggests comparable effectiveness between the combined method and the low‐dose vaginal misoprostol alone. The safety of using a balloon catheter with concurrent low‐dose vaginal misoprostol remains uncertain. Given the limited available data and the outstanding safety questions, the combined method cannot be recommended for routine clinical decision making in the present stage. The results could slightly favor the combined method due to reduced hyperstimulation and meconium‐stained liquor.

## AUTHOR CONTRIBUTIONS

MP principally managed the project and the collaborative process and carried out design, data collection, IPD checking, data synthesis, writing, editing, finalizing, and submitting of the manuscript. BWM and WL conceived the research ideal and were responsible for overseeing all aspects of conduct. FC and WL contributed to eligibility screening and risk of bias assessment as the second and third reviewers. DLR, and BWM provided clinical oversight. MP designed and conducted the literature searches. All authors were involved in the decision to submit the manuscript. All contributing trial investigators had opportunities to comment on the initial scope, draft protocol and draft statistical analysis plan and participated in e‐discussions as the project progressed. Trial investigators also prepared and supplied data and answered questions about their RCTs.

## FUNDING INFORMATION

This study was supported by three NHMRC Investigator Grants (GNT1176437 for BWM, GNT2016729 for WL, and GNT 2025670 for DLR). These funding sources had no role in the design, execution, analyses, or data interpretation of this research. MP is supported by a Research Training Stipend provided by the Australian Government.

## CONFLICT OF INTEREST STATEMENT

MP is supported by a Research Training Stipend, provided by the Australian Government. BWM declared grants from NHMRC, personal fees from ObsEva, personal fees from Merck, personal fees from Guerbet, other from Guerbet, and grants from Merck, outside the submitted work. DLR declared grants from NHMRC and has received travel support and lecture fees from the International Society of Ultrasound in Obstetrics and Gynecology (ISUOG), unrelated to this work. WL declared a grant from NHMRC that supports this work. The remaining authors report no conflict of interest.

## Supporting information


Data S1.

## Data Availability

The protocol, statistical analysis plan and codebook are available on request. The trial investigators who shared individual participant data for the purposes of the meta‐analysis retain ownership of their trial data and any requests for access to individual participant data should be made directly to them.
