# Supplementary material for: Balloon catheter plus concurrent vaginal misoprostol versus vaginal misoprostol alone for cervical ripening in induction of labor: A systematic review and an individual participant data meta‐analysis of randomized controlled trials
Source: Int J Gynaecol Obstet. 2026 Mar 12;174(3):1188–204. doi: 10.1002/ijgo.70949 (PMC13505249; doi:10.1002/ijgo.70949)
Supplement: Supplementary file 1 [file IJGO-174-1188-s001.zip › IPD 3 Supplementary Figures and Table FIGO IJGO_V5.docx]

**Supplementary Files: Figures and Tables**

**Title:** Balloon catheter plus concurrent vaginal misoprostol versus vaginal misoprostol alone for cervical ripening in induction of labor: individual participant data meta-analysis of randomized controlled trials.

**Supplementary Figures**

Supplementary Figure 1: Risk of bias assessment of both participating and non-participating randomized controlled trials.

1A

1B

1A. Risk of bias assessment of IPD shared (‘*participating*’) randomized controlled trials. 1B. Risk of bias assessment of IPD non-shared (‘*not participating’*) randomized controlled trials.

IPD: Individual participant data.

**Supplementary Figures 2 - 12 show the secondary outcomes results for the balloon catheter plus concurrent low-dose vaginal misoprostol (combined method) versus low-dose vaginal misoprostol alone for cervical ripening in induction of labor.**

Supplementary Figure 2. Odds of cesarean deliveries (two-stage meta-analysis).

Supplementary Figure 3. Odds of cesarean deliveries for failure to progress (two-stage meta-analysis).

Supplementary Figure 4. Odds of unassisted vaginal deliveries (two-stage meta-analysis).

Supplementary Figure 5. Odds of instrumental vaginal birth (two-stage meta-analysis).

Supplementary Figure 6. Time to vaginal birth (two-stage meta-analysis).

IDI: Induction to delivery interval; HR: Hazard ratio

Supplementary Figure 7. Oxytocin use (two-stage meta-analysis).

Supplementary Figure 8. Meconium-stained amniotic fluid (two-stage meta-analysis).

Supplementary Figure 9. Use of epidural analgesia (two-stage meta-analysis).

Supplementary Figure 10. Maternal infections (two-stage meta-analysis).

Supplementary Figure 11. Arterial umbilical cord pH < 7.10 (two-stage meta-analysis).

Supplementary Figure 12. Admission to neonatal intensive care unit (two-stage meta-analysis).

Supplementary Figure 13. Funnel plot.

**Supplementary Tables**

Supplementary Table 1. Search Strategies.

| **Source** | **Search** |
| --- | --- |
| Ovid Medline/ Ovid EMBASE/ Ovid Emcare | Labo?r ADJ2 (induct* OR induce*) \| Bishop* score \|  Labor, Induced/ OR [Cervical Ripening](https://ovidsp.dc2.ovid.com/ovid-b/ovidweb.cgi?&Controlled+Vocabulary=Mapping%7c6&Return=mapping&S=EEGNFPIOHMEBKKENIPNJHFAFECFCAA00)/ \|  [Cervi* Ripening](https://ovidsp.dc2.ovid.com/ovid-b/ovidweb.cgi?&Controlled+Vocabulary=Mapping%7c6&Return=mapping&S=EEGNFPIOHMEBKKENIPNJHFAFECFCAA00) OR Cervi* priming or Pre-induction OR Preinduction\|  Balloon OR Balloon catheter Single balloon* OR single-balloon* OR Double balloon* OR double-balloon* OR Foley* OR Atad* OR Cook* OR Bard* \|  AND Misoprostol/ |
| Cochrane Library  (Trials only) | Labo?r ADJ2 (induct* OR induce*)\|  [Cervi* Ripening](https://ovidsp.dc2.ovid.com/ovid-b/ovidweb.cgi?&Controlled+Vocabulary=Mapping%7c6&Return=mapping&S=EEGNFPIOHMEBKKENIPNJHFAFECFCAA00) or Cervi* priming or Pre-induction or Preinduction) \|  Balloon or Balloon catheter OR Single balloon* OR Single-balloon* OR Double balloon* OR Double-balloon* OR Foley* OR Atad* OR Bard* OR Misoprostol\|    AND Misoprostol |
| ClinicalTrials.gov | Condition or disease: induction of labor OR cervical ripening OR cervical priming OR cervix priming OR preinduction OR pre-induction OR (labor NOT work) OR (labour NOT work)  Other terms: Balloon or Balloon catheter OR Foley* OR Cook* AND Misoprostol OR Prostaglandin E1 OR PGE1 |
| WHO ICTRP | Labour induction or cervical ripening AND Balloon OR Balloon catheter OR Foley AND Misoprostol |
| Scopus | (TITLE-ABS-KEY("Induc*" W/2 labo?r) OR TITLE-ABS-KEY(bishop* AND score) OR TITLE-ABS-KEY(pre-induction) OR TITLE-ABS-KEY(preinduction) OR TITLE-ABS-KEY(cervi* AND ripening) OR TITLE-ABS-KEY(cervi* AND priming) OR TITLE-ABS-KEY(( foley* OR cook* OR atad ) W/2 ( balloon OR catheter )) OR TITLE-ABS-KEY("Double balloon") OR TITLE-ABS-KEY("Single balloon") AND TITLE-ABS-KEY("Prostaglandin E1") AND TITLE-ABS-KEY(misoprostol) AND ALL(randomi?ed AND controlled AND trial* AND controlled AND clinical AND trial*)) |

Supplementary Table 2. Definition of outcomes and potential effect modifiers as presented in the prospectively registered study protocol.

**PRIMARY OUTCOMES**

**Vaginal delivery rate:** rate of vaginal delivery following randomization per woman randomised.

**Composite of adverse perinatal outcome:** a composite measure of stillbirth, neonatal death, neonatal Apgar score <7 at 5 minutes, acidosis (pH<7.1), neonatal seizures, hypoxic ischemic encephalopathy of any stage (HIE), neonatal intensive care unit (NICU) admission, meconium aspiration syndrome^¶^, neonatal infection either clinically suspected (as defined by neonatal antibiotic administration) or proven neonatal infection (culture proven), cord prolapse, endotracheal intubation and/or external cardiac compressions.

**Composite of adverse maternal outcome:** maternal infection (temperature ≥ 38°C at any time during labor or delivery, antibiotics use or clinically diagnosed infection, such as endometritis), maternal admission to intensive care unit, severe postpartum hemorrhage (>1000mL estimated blood loss), uterine rupture or maternal death.

**SECONDARY OUTCOMES**

**Delivery outcome:**

- Cumulative rate of vaginal birth (time-to-event analysis)
- Time from commencement of labor induction to delivery
- Mode of delivery (cesarean delivery, unassisted vaginal birth, instrumental vaginal birth)
- Cesarean delivery, including analysis for fetal compromise or failure to progress (if both fetal compromise and failure to progress apply as indications, fetal compromise prevails).
- Indication for instrumental vaginal birth
- Total hospital stays for the mother and neonate*

**Labor progression outcomes:**

- Uterine tachysystole
- Uterine hyperstimulation
- Oxytocin augmentation
- Meconium-stained amniotic fluid
- Use of epidural analgesia
- Use of more methods for cervical ripening (second methods)

**Neonatal safety outcomes:**

- Cord prolapse
- Apgar score <7 at five minutes
- Arterial umbilical cord pH <7.10
- Neonatal intensive care unit admission

**Maternal safety outcomes:**

- Maternal infection
- Severe postpartum hemorrhage (>1000mL estimated blood loss)
- Maternal admission to the intensive care unit
- Other complications requiring hospital assistance^#^

**Maternal satisfaction:**

**POTENTIAL EFFECT MODIFIERS**

- Maternal parity
- Maternal age
- Maternal body mass index (BMI)
- Initial Bishop score
- Indication for induction of labor
  - Post-dated gestation
  - Hypertensive disorders
  - Diabetes or gestational diabetes*
  - Intra-uterine/fetal growth restriction*
  - Oligohydramnios*
  - Obstetric cholestasis*
  - Advanced maternal age*
  - Elective/maternal request*
  - Multiple indications*

All intended outcomes are listed in the statistical analysis plan. ^¶^Neonatal pneumonia resulting from aspiration of meconium. ^#^ We intended to examine complications requiring maternal hospital assistance; however, this was not readily available in the trials included. *Insufficient data were available to perform the planned analysis.

Supplementary Table 3: Distribution of TRACT trustworthiness ratings across seven domains among IPD non-shared randomized controlled trials identified as ‘not meeting trustworthiness criteria’.

| *DOMAIN (1)* | *ITEM* | *TRACT RATING** | | | | | | | | | |
| --- | --- | --- | --- | --- | --- | --- | --- | --- | --- | --- | --- |
|  |  | IPD non-shared randomized controlled trials | | | | | | | | | |
|  |  | Bhatiyani 2017 | Ashwini 2018 | El-Kelani 2019 | Kareem 2020 | Aregeb 2021 | Devi 2021 | Izevbigie 2021 | Sharma 2021 | Elpo 2022 | Toshniwal 2022 |
| Governance | Absent or retrospective registration of RCTs. This is relevant for RCTs commencing after 2010 | Major concerns | Major concerns | Major concerns | Major concerns | Major concerns | Major concerns | Major concerns | Major concerns | Major concerns | Major concerns |
|  | Discrepancy of >15% between the intended sample size in the trial registration compared to the actual sample size achieved in the RCT | Some Concerns/No Information | Some Concerns/No Information | Some Concerns/No Information | Some Concerns/No Information | Some Concerns/No Information | Some Concerns/No Information | Some Concerns/No Information | Some Concerns/No Information | Major concerns | Some Concerns/No Information |
|  | Absent or vague description of research ethics or apparent concerns regarding ethics | Major concerns | Some Concerns/No Information | Some Concerns/No Information | Some Concerns/No Information | Some Concerns/No Information | Some Concerns/No Information | Major concerns | Major concerns | Some Concerns/No Information | Major concerns |
| Author Group | Number of authors $\leq$3 or low author to study size ratio | No Concerns | Major concerns | No Concerns | No Concerns | No Concerns | No Concerns | No Concerns | No Concerns | Major concerns | Major concerns |
|  | Other studies of authors have been retracted not on request of the authors | No Concerns | No Concerns | No Concerns | No Concerns | No Concerns | No Concerns | No Concerns | No Concerns | No Concerns | No Concerns |
|  | Large number of RCTs published in a short time frame by one author/in one institute | No Concerns | No Concerns | No Concerns | No Concerns | No Concerns | No Concerns | No Concerns | No Concerns | No Concerns | No Concerns |
| Plausibility of Intervention Usage | Insufficient or implausible description of allocation concealment (e.g. two interventions but only one placebo) | Some Concerns/No Information | No Concerns | Some Concerns/No Information | Some Concerns/No Information | Some Concerns/No Information | Some Concerns/No Information | Some Concerns/No Information | Some Concerns/No Information | Some Concerns/No Information | Some Concerns/No Information |
|  | Unnecessary or illogical description of methodological standards (e.g. use of sealed envelopes in a placebo-controlled trial) | No Concerns | No Concerns | No Concerns | No Concerns | No Concerns | No Concerns | No Concerns | No Concerns | No Concerns | No Concerns |
| Timeframe | Fast recruitment of participants within the study time (especially single centre studies) | No Concerns | No Concerns | No Concerns | No Concerns | No Concerns | No Concerns | No Concerns | No Concerns | Some Concerns/No Information | No Concerns |
|  | Short or impossible time frame between ending recruitment/follow up and submission of the paper (take into account time to outcome | No Concerns | No Concerns | No Concerns | No Concerns | No Concerns | No Concerns | No Concerns | No Concerns | Some Concerns/No Information | No Concerns |
| Drop-Out Rates | Zero participants lost to follow up or no reasons mentioned for loss of follow up | Major concerns | Major concerns | Major concerns | Major concerns | No Concerns | Major concerns | Major concerns | Major concerns | No Concerns | Major concerns |
|  | Ideal number of losses to follow up resulting in perfectly rounded number in each group | Major concerns | Major concerns | Major concerns | Major concerns | No Concerns | Major concerns | Major concerns | Major concerns | No Concerns | Major concerns |
| Baseline Characteristics | No or few baseline (<5) characteristics presented | Major concerns | Major concerns | Major concerns | No Concerns | Major concerns | Major concerns | No Concerns | Major concerns | No Concerns | Major concerns |
|  | Implausible patient characteristics judging from common sense, the literature and local data (e.g. similar standard deviations for completely different characteristics with different means and distributions) | Some Concerns/No Information | Some Concerns/No Information | Some Concerns/No Information | Some Concerns/No Information | Some Concerns/No Information | Some Concerns/No Information | No Concerns | Some Concerns/No Information | No Concerns | Some Concerns/No Information |
|  | Perfect balance for multiple baseline characteristics or significant/large differences between baseline characteristics | Some Concerns/No Information | Some Concerns/No Information | Some Concerns/No Information | Some Concerns/No Information | Some Concerns/No Information | Some Concerns/No Information | No Concerns | Some Concerns/No Information | No Concerns | Some Concerns/No Information |
|  | Important prognostic factors are not reported as baseline characteristics | Major concerns | Major concerns | Major concerns | No Concerns | Major concerns | Major concerns | No Concerns | Major concerns | No Concerns | Major concerns |
| Outcomes | Effect size that is much larger than in other RCTs regarding the same topic | No Concerns | Some Concerns/No Information | No Concerns | No Concerns | No Concerns | No Concerns | No Concerns | No Concerns | No Concerns | No Concerns |
|  | Conflicting information between outcomes | No Concerns | No Concerns | No Concerns | No Concerns | No Concerns | No Concerns | No Concerns | No Concerns | No Concerns | No Concerns |
|  | Change in primary outcome from registration to publication | Some Concerns/No Information | Some Concerns/No Information | Some Concerns/No Information | Some Concerns/No Information | Some Concerns/No Information | Some Concerns/No Information | Some Concerns/No Information | Some Concerns/No Information | Some Concerns/No Information | Some Concerns/No Information |
| Total TRACT Score** | | 17 | 18 | 16 | 12 | 12 | 16 | 11 | 17 | 11 | 19 |

*TRACT: Trustworthiness in RAndomised Controlled Trials (TRACT checklist); RCT: Randomised controlled trial. **We adopted a scoring system for this TRACT checklist (‘*No Concerns*’ - 0 scores, ‘*Some Concerns/No Information*’ - 1 score, ‘*Major concerns’* - 2 scores). The categorisation of 'not meeting our trustworthiness criteria' and ‘meeting our trustworthiness criteria’ for RCTs was based on the on the overall quality of the paper across all seven domains, published year, availability of raw data and concerns over critical items on the TRACT checklist.

1. Mol BW, Lai S, Rahim A, Bordewijk EM, Wang R, van ER, et al. Checklist to assess Trustworthiness in RAndomised Controlled Trials (TRACT checklist): concept proposal and pilot. Res Integr Peer Rev. 2023;8(1):6.

Supplementary Table 4. Treatment-covariate interaction for vaginal birth.

| **Effect modifier** | **No. of trials** | **No. of women** | **aOR (95% CI), p-value for interaction** | **I^2^** |
| --- | --- | --- | --- | --- |
| Parity | 5 | 649 | 0.63 (0.26;1.50), p = 0.21 | 0 |
| Maternal age | 5 | 649 | 0.98 (0.89;1.08), p = 0.57 | 0 |
| BMI | 3 | 414 | 1.02 (0.72;1.45), p = 0.81 | 49 |
| Initial Bishop score | 3 | 414 | 1.20 (0.50;2.86), p = 0.47 | 16 |
| Gestational age | 5 | 649 | 1.06 (0.69;1.64), p = 0.72 | 18 |
| Indication for IOL*^#^ |  |  |  |  |
| - Hypertensive disorders | 5 | 105 | 0.84 (0.51;1.40), p = 0.40 | 0 |
| *All compared to induction of labour for post-dates (reference) | | | | |

aOR: adjusted odds ratio; 95% CI: 95% Confidence Interval; IOL: Induction of labor; BMI: Body mass index

^#^Data were insufficient for diabetes mellitus, fetal growth restriction, oligohydramnios, obstetric cholestasis, advanced maternal age, elective/maternal request and multiple indications.
