# Supplementary material for: Balloon catheter plus concurrent vaginal misoprostol versus vaginal misoprostol alone for cervical ripening in induction of labor: A systematic review and an individual participant data meta‐analysis of randomized controlled trials
Source: Int J Gynaecol Obstet. 2026 Mar 12;174(3):1188–204. doi: 10.1002/ijgo.70949 (PMC13505249; doi:10.1002/ijgo.70949)
Supplement: Supplementary file 1 [file IJGO-174-1188-s001.zip › Appendix A - SAP IPDMA 3 Combined methods 3-8-2024 V1.pdf]

Statistical Analysis Plan IPD- Balloon catheter plus concurrent low-dose vaginal misoprostol versus low-dose vaginal misoprostol alone for induction of labour.

Title

Balloon catheter plus concurrent low-dose vaginal misoprostol versus low-dose vaginal misoprostol alone for induction of labour: individual participant data meta-analysis of randomised controlled trials.

Members

M Patabendige, D.L. Rolnik, B.W. Mol, W. Li

Department of Obstetrics and Gynaecology, School of Clinical Sciences at Monash Health, Monash University, Clayton, VIC, Australia.

Contents

Introduction ... 3
Rationale ... 3
Objectives ... 4
Methods ... 4
Search strategy and eligibility criteria ... 4
Data collection ... 6
Software ... 6
STATISTICAL ANALYSIS I: META-ANALYSIS ... 6
Risk of bias, quality appraisal and data checking ... 7
Overall approach to IPD meta-analysis ... 8
Type of analysis ... 8
Protocol violations, withdrawals, missing data, outliers and unexpected irregularities ... 9
Baseline characteristics ... 12

Treatment outcomes 13
Effect modifiers (treatment-covariate interaction) 21
STATISTICAL ANALYSIS II: META-ANALYSIS 23
STATISTICAL ANALYSIS III: SUBGROUP ANALYSIS 24
STATISTICAL ANALYSIS III: SENSITIVITY ANALYSIS 24

PROTOCOL SIGNATURE SHEET

**Name**	**Signature**
Investigator<br>Malitha Patabendige	
Investigator<br>Wentao Li	
Investigator<br>Daniel L. Rolnik	
Principal investigator<br>Ben W. Mol	

Introduction

Rationale

Induction of labour (IOL) is an increasingly common obstetric procedure used to stimulate the commencement of labour (1, 2). It is indicated when the benefits of delivery outweigh the risks of continuing the pregnancy to the mother or the fetus (2). Methods for inducing labour include mechanical means (e.g. balloon catheter) or pharmacological means (e.g. vaginal misoprostol, a synthetic prostaglandin E1) (3). Balloon catheters work by giving physical pressure on the cervix, which stimulates the release of prostaglandins locally to act on the cervical smooth muscle (4). Compared to pharmacological methods, the Foley catheter has an additional benefit of a decreased risk of causing uterine hyperstimulation (4). However, pharmacological methods have been reported to reduce the time from induction to delivery (5, 6). This balance of safety and efficacy has prompted the concept of combined mechanical and pharmacological cervical ripening and IOL (7).

An individual participant data (IPD) meta-analysis allows for comparing two interventions using the raw data collected by investigators who conducted the original trials. This collation of patient data can subsequently be combined and subjected to analyses to allow consistent comparison and assessment of the results (8). By collating the data, a larger sample size of patients is obtained. IPD also allows for subgroup analysis, permitting more individualised results, which can be applied to a specific population. Conducting an IPD meta-analysis allows for coherent comparisons of the interventions across all studies involved, providing greater insight into the efficacy and safety behind different labour induction methods. There is emerging interest and evidence on the use of combined mechanical and pharmacological methods for IOL. As per the recent Cochrane review on mechanical methods for IOL (2), we only chose to include low-dose misoprostol (defined as ≤ 50 mcg every ≥ 4 hours) as evidence suggests low-dose misoprostol is superior to high-dose misoprostol regarding safety outcomes and being equally effective.

.

This is an IPDMA of randomised control trials (RCTs) that compare the effectiveness and safety of the cervical ripening and IOL with balloon catheter plus concurrent low-dose vaginal misoprostol compared with low-dose vaginal misoprostol alone.

Objectives

1. To compare the effectiveness, perinatal and maternal safety of induction of labour with balloon catheter plus concurrent low-dose vaginal misoprostol compared with low-dose vaginal misoprostol alone.
2. To study differences in the effectiveness of induction of labour with balloon catheter plus concurrent low-dose vaginal misoprostol compared with low-dose vaginal misoprostol alone with:
   A. Nulliparous or multiparous
   B. Various maternal age
   C. Various maternal BMI
   D. Various initial Bishop score
   E. Different indications for induction of labour

Methods

<u>Search strategy and eligibility criteria</u>

RCTs with balloon catheter plus concurrent low-dose vaginal misoprostol compared with low-dose vaginal misoprostol alone for third trimester (≥ 34 weeks) labour induction will be included. Women with prelabour rupture of membranes and trial of scar will also be included. Trials with high dose misoprostol, cluster randomised trials and quasi-experimental studies will be excluded.

Potentially suitable clinical trials will be identified from inclusion in the Cochrane Review on Mechanical Methods for Induction of Labour (2019). Databases, including Ovid MEDLINE,

Embase via Ovid, Ovid Emcare, Scopus, Cochrane Pregnancy and Childbirth Group's Trials Register, WHO International Clinical Trials Registry Platform (ICTRP), clinicaltrials.gov (for unpublished, planned and ongoing trial reports), and reference lists of retrieved studies will be searched to identify further trials published since the above 2019 Cochrane Review was conducted. This database search was conducted for the gap from the above Cochrane review search and was updated in July 2024. There will be no language barriers and all the published and unpublished data will be eligible. As per the recent Cochrane review on mechanical methods for IOL (2), we only chose to include low-dose misoprostol (defined as ≤ 50 mcg every ≥ 4 hours) as evidence suggests low-dose misoprostol is superior to high-dose misoprostol regarding safety outcomes and being equally effective.

Two investigators will independently review the identified papers for eligibility, with disagreements to be solved by a third reviewer. The principal authors of eligible trials will be invited to contribute their raw data for analysis. An IPDMA will be performed on available data to assess the impact on primary and secondary outcome measures, as outlined below.

Population

Pregnant women in the third trimester (≥ 34 weeks) with a live, singleton fetus and with an unfavourable cervix who require cervical ripening in induction of labour.

Intervention/treatment

The intervention measured in each study was balloon catheter plus concurrent low-dose vaginal misoprostol for cervical ripening in induction of labour.

Comparator/control

The comparator was the use of low-dose vaginal misoprostol alone for third trimester labour induction.

Outcomes

Studies were eligible if they reported the rate of vaginal delivery for each group.

Data collection

The principal investigators of all eligible trials identified were contacted via email to participate in this study. If there was no response for two weeks, another gentle reminder was emailed. They were asked to share the individual patient data collected from their trials. If they failed to respond, the co-authors, last authors, institute and journal editors were contacted via email. The principal investigators were asked to complete an Excel datasheet or send their data with a document clearly stating the definitions used for variables. The collected data should contain baseline patient characteristics including age, gestational age, parity, ethnicity, body mass index (BMI) as pre-pregnancy and/or at the time of randomisation, smoking status, and the modified Bishop score before intervention. Coding of the data guaranteed that the key to linking IPD to patient identity remained in the possession of each research group. For those studies for which IPD cannot be obtained, we sought to collect the reasons for the non-availability of IPD.

Software

SPSS and R will be used for all statistical analyses.

STATISTICAL ANALYSIS I: META-ANALYSIS

Flow chart

We will construct the PRISMA-IPD flow diagram(9) detailing the process of study selection and collection of IPD and aggregate data. This diagram will report the number of studies identified, screened for eligibility, and included or excluded, together with the number of participants in these studies and the reasons for exclusion at each stage. The flow diagram will also report the number of studies that provided IPD and those that did not, along with the reasons why IPD could not be

obtained in the latter case and the number of participants for which IPD could or could not be obtained.

Risk of bias, quality appraisal and data checking

- Risk of bias

Two review authors will independently assess study quality and risk of bias. If there are disagreements, a third author will be consulted. The risk of bias will be assessed using Version 2 of the Cochrane risk-of-bias tool for randomised trials (RoB 2) (10). Additionally, we will test for data availability bias by constructing the summary effect estimates with all the RCTs.

- Quality of the data, Integrity Check and trustworthiness assessment

Data will be checked for missing values and entries that are outside the expected ranges, and internal consistency checks will be performed. The pattern of missing data will be analysed separately for each dataset using the patterns chart. All variables besides the ID variable will be selected. We will analyse variables that are missing >0.01% of data. First, we will check which variables have missing data. Then, using the missing value pattern chart, we will look for monotonicity. If missing data is concentrated in the upper left and lower right corner, there is a systematic pattern in missing data. If there are only patches or islands of missing values, the data is likely missing at a random pattern.

The quality of the data will be assessed by comparing the provided IPD with the published baseline data and results. The baseline table will be reconstructed for each study. The outcome data will be analysed, and the results will be compared with the published analyses. In case of any discrepancies or queries which may arise, the principal investigator will be contacted, and

corrections will be made if necessary. Key design features and demographic characteristics of the included trials will be summarised in a table.

We will also check the trustworthiness concerns on the studies using the Trustworthiness in RAndomised Controlled Trials (TRACT) trustworthiness checklist (11). This screening tool aims to help identify and triage studies at risk of integrity issues. The checklist includes seven domains that apply to every study: governance, author group, plausibility of intervention usage, timeframe, drop-out rates, baseline characteristics and outcomes. Two review authors will perform the assessment for all the studies published in full-text form and disagreements will be resolved by consensus. We adopted a scoring system for this TRACT checklist (‘*No Concerns*’ - 0 score, ‘*Some Concerns/No Information*’ - 1 score, ‘*Major concerns’* - 2 scores) to make it easier for the categorisation. The categorisation of *'not meeting our trustworthiness criteria'* and ‘*meeting our trustworthiness criteria’* for studies will be based on the TRACT score and the overall quality of the paper.

Overall approach to IPD meta-analysis

Our primary analysis will be a ‘two-stage’ meta-analysis method to synthesise the IPD. This will be conducted to obtain forest plots of the effect estimates and a measure of between-study heterogeneity. However, for outcomes that had zero events in any intervention group in any included trial, a ‘one-stage’ method will be used. A ‘one-stage’ meta-analysis will also be performed for primary outcomes as a sensitivity analysis strategy.

Type of analysis

Primarily, an intention to treat analysis will be performed. An as treated (AT) analysis will be carried out as part of a sensitivity analysis strategy.

- ➢ Intention to treat (ITT): All participants will be included in the analysis and will be evaluated according to the treatment for which they were randomised.

- ➢ As treated (AT): All participants will be analyzed according to the treatment they actually received. If participants became lost to follow-up or withdrew their informed consent before the intervention could have taken place, they will be excluded from all analyses.

Protocol violations, withdrawals, missing data, outliers and unexpected irregularities

Criteria

- ➢ **Protocol violations:**
- ✓ Wrongfully included patients will be excluded from the full analysis set.
- ✓ Cross-over: patients receiving the treatment of the arm to which they were not randomised will primarily be analysed according to the treatment to which they were allocated (i.e. intention to treat analysis). In the secondary as treated analysis, these patients will be analysed in the arm corresponding to the treatment they actually received.

- ➢ **Withdrawals:**
- ✓ Withdrawals will be included in the data analysis up to when the participant withdrew informed consent.
- ✓ Missing covariate data after withdrawal of informed consent will be marked as 'missing' and imputed.
- ✓ Participants with missing outcome data after withdrawal of informed consent will be excluded from analysis for the missing outcome(s).

- ➢ **Lost to follow-up:**
- ✓ Data will be included until the time at which the participant became 'lost to follow up' during the follow-up period of the study.

- ✓ Missing covariate data for participants who became lost to follow-up will be marked as ‘missing’ and imputed.
- ✓ Participants with missing outcome data after loss to follow-up will be excluded from analysis for the missing outcome(s).

- ➢ **Missing data:**
- ✓ No available data at all for a participant: exclusion from the full analysis set.
- ✓ Missing outcome data: exclusion from analysis for the missing outcome(s).
- ✓ Sporadically missing covariate data: multiple imputation.
- ✓ Entire variables missing from one study (systematically missing data): no imputation.
- ✓ For further details on imputation see <u>Methods for imputation of missing data</u> below.

- ➢ **Unexpected irregularities:**
- ✓ The strategy will be decided on when an unexpected irregularity is found in the dataset. This will be clearly described in the final manuscript.

- ➢ **Outliers:**
- ✓ If outliers cannot be resolved, they will be marked as ‘missing’ and imputed.

The abovementioned is summarized in the following table:

Category	Problem	Action
Protocol violation	Wrongfully included patients	Exclude from all analyses
	Cross-over	ITT analysis: analyze according to allocation<br>AT analysis: analyze according to received<br>treatment
Withdrawals<br>***And loss to follow-up***	Before the time point for which intervention<br>was scheduled	Exclude from all analyses
	After the time point for which intervention was scheduled	Include in all analyses of outcomes for which outcome data is available
Missing data	No available data at all	Exclude
	Sporadically/randomly missing data	Determine likely missing pattern<br>Multiple imputation within studies if MAR
	Systematically missing data	No imputation
Unexpected irregularities	Not yet known	To be determined on a case-by-case basis
Outliers	Outliers will primarily be tried to resolve. It may sometimes be impossible to resolve.	Mark as missing and impute

Criteria for handling protocol violations, missing data, outliers and unexpected irregularities in the datasets. Abbreviations: MAR = missing at random

Methods for imputation of missing data

- Only missing covariate data will be imputed with multiple imputation. No outcome will be imputed.
- Sporadically missing data will be imputed *within each study* by means of chained equations (10 datasets).
- Multiple imputation techniques assume MAR (missing at random) pattern. If data is likely to be MNAR (missing not at random) or MCAR (missing completely at random), we will conduct a sensitivity analysis in order to assess the robustness of the imputed datasets under different hypotheses for the missingness pattern.

Baseline characteristics

Methods

- Baseline tables will be constructed separately for each study and will display only the baseline characteristics of those participants included in the analysis.
- In all cases where we aim to report a mean (±SD), a median with interquartile ranges will be given instead of mean with standard deviation if the variable is non-normally distributed. Binary and categorical variables will be presented as the number of total and percentage.
- No statistical test will be performed to determine if the groups are significantly different from each other or not, as we assume any baseline differences after randomisation have occurred due to chance.
- Overall baseline characteristics will be presented per group.

Missing values

- The number of missing values will be indicated in the table.

Variables

- The following variables will be reported:

1. Maternal age in years
2. Maternal Body Mass Index in kg/m2
3. Female parity, n (%)

➢ 0

➢ 1

➢ ≥2

4. Indication for induction of labour

➢ Hypertensive disorders, n (%)

➢ Post-dates gestation, n (%)

➢ Diabetes/gestational diabetes, n (%)

➢ Intrauterine growth restriction/fetal growth restriction, n (%)

➢ Oligohydramnios, n (%)

➢ Advanced maternal age, n (%)

➢ Elective, n (%)

➢ More than one indication, n (%)

➢ Other/unknown, n (%)

5. Initial Bishop score

Treatment outcomes

We will study the effect of treatment for the following outcomes:

<u>Primary:</u>

1. Vaginal delivery rate

2. Composite of adverse perinatal outcome: a composite measure of stillbirth, neonatal death, neonatal Apgar score <7 at 5 minutes, acidosis (pH<7.1), neonatal seizures, hypoxic ischaemic

encephalopathy of any stage (HIE), neonatal intensive care unit (NICU) admission for any duration, meconium aspiration syndrome, neonatal infection either clinically suspected (as defined by neonatal antibiotic administration) or proven neonatal infection (culture proven), endotracheal intubation and/or external cardiac compressions.

3. Composite of adverse maternal outcome: a composite measure of admission to ICU for any period, maternal infection (defined as a temperature ≥ 38°C at any time during labour or delivery or antibiotic use or clinically diagnosed infection, such as endometritis), postpartum haemorrhage ≥1000 mL, maternal death, uterine rupture.

Secondary:

1. Delivery outcomes:

- Mode of delivery (spontaneous vaginal birth, instrumental vaginal birth and caesarean section)

- Caesarean section indications (i.e., fetal compromise or failure to progress. If both fetal compromise and failure to progress apply as indications, fetal compromise will prevail.

- Instrumental vaginal birth and indication for instrumental vaginal birth- fetal compromise or failure to progress. If both fetal compromise and failure to progress apply as indications, fetal compromise will prevail.

- Time from commencement of labour induction to vaginal delivery (cumulative rate of vaginal birth)

2. Labour progression outcomes:

- Uterine tachysystole, hypertonus
- Uterine hyperstimulation

- Oxytocin augmentation
- Meconium-stained amniotic fluid
- Use of epidural analgesia and opioid analgesia during labour

3. Maternal safety outcomes:
- Individual components of the primary composite maternal outcome

4. Neonatal safety outcomes
   - Individual components of the primary composite perinatal outcome

5. Satisfaction
   - Maternal satisfaction

Additionally, other parameters such as neonatal birth trauma (mechanical causes during childbirth), bowel obstruction, thrombo-embolic events, stroke, hypertensive disorders, hysterectomy for any complications resulting from birth, Damage to internal organs (bladder, bowel, ureters), cardio-respiratory arrest and other maternal postpartum conditions requiring treatment will be assessed. However, these will be analysed depending on the availability of adequate data.

The way outcomes are recorded, and their corresponding variables and possible values are summarized in the following table:

Intervention (Group A) - balloon catheter plus concurrent low-dose vaginal misoprostol.

Reference/Control (Group B) - vaginal misoprostol. FHR: Fetal heart rate; MOD: Mode of delivery.

Identification	**my_ID** (my participant ID)	Continuous

	**Original_ID**<br>(old participant ID)	Continuous<br>Study ID used in the original study
	**author**	RCT name_year
	**RCT**	Categorical
Induction category	**ind_cat**<br>induction category	Categorical<br>0 = vaginal misoprostol<br>1 = combined
	**Singleton**<br>Fetal number	Categorical<br>0 = singleton, 1 = multiple
Baseline variables	**Age** years	Continuous
	**Weight** /kg	Continuous
	**Height**/cm	Continuous
	**bmi** kg/m2	Continuous
	**gesta_weeks**<br>(gestational age in weeks)	Continuous
	ethnicity	Categorical<br>0 = Non-Caucasian<br>1 = Caucasian
	smoking<br>(Smoking status)	Categorical<br>0 = no, 1 = yes
	**parity**	Continuous
	**parcode**<br>(parity code)	Categorical<br>0 = nulliparous<br>1 = multiparous
	**Ind_iol** (indication for IOL)	
	Categorical<br>0 = other/unknown	

	1 = hypertensive disorders<br>2 = postdates<br>3 = diabetes/gestational diabetes<br>4 = IUGR/fetal growth restriction<br>5 = oligohydramnios<br>6 = advanced maternal age<br>7 = obstetric cholestasis<br>8 = elective/social<br>9 = >1 indication	
**bs_initial**<br>(initial Bishop score before intervention)		Continuous
**bs_max**<br>(Maximum Bishop score achieved)		Continuous
**srom**<br>Membrane status		Categorical<br>1= intact<br>0= ruptured
Primary and secondary delivery outcomes	**mod**<br>(mode of delivery)	Categorical<br>2 = instrumental<br>1 = caesarean section<br>0 = vaginal unassisted
	**Instr_ftp**<br>Instrumental delivery for failure to progress	Categorical<br>0 = no, 1 = yes
	**Instr_fhr**<br>instrumental delivery for abnormal FHR	Categorical<br>0 = no, 1 = yes
	**Cs_ftp_new**<br>Caesarean section for failure to progress	Categorical<br>0 = no, 1 = yes
	**Cs_abn_ftr**<br>Caesarean section for abnormal FHR	Categorical<br>0 = no, 1 = yes
	failed_induction (use of other methods for IOL after originally allocated method)	Categorical<br>0 = no, 1 = yes

	**Idi_hr** (induction to delivery interval in hours)	Continuous
	**Idi_min** (induction to delivery interval in min)	Continuous
	**ut_tachy**<br>(uterine tachysystole	Categorical<br>0 = no, 1 = yes
	**ut_ hypertonus**<br>hypertonus	Categorical<br>0 = no, 1 = yes
	**ut_hypersti**<br>(uterine hyperstimulation)	Categorical<br>0 = no, 1 = yes
	Oxy_aug<br>(use of oxytocin during labour)	Categorical<br>0 = no, 1 = yes
Secondary labour progression outcomes	**Mec_amnio**<br>(meconium stained amniotic fluid)	Categorical<br>0 = no, 1 = yes
	**analge**<br>(analgesia during labour)	Categorical<br>0 = no, 1 = yes
	**epidural**<br>Epidural use	Categorical<br>0 = no, 1 = yes
	**analge_cat**<br>type_analgesia<br>(type of analgesia during labour)	Categorial<br>0 = None<br>1 = Parental opioids<br>2 = Epidural use<br>3 = Entonox<br>4 = Other
	Oxy_duration (duration of oxytocin infusion in hours)	Continuous
	**m_anbx**<br>(maternal antibiotics given)	Categorical<br>0 = no, 1 = yes
	**m_fever** (maternal temperature ≥38C)	Categorical<br>0 = no, 1 = yes

	**m_infec** (suspected/proven maternal infection)	Categorical<br>0 = no, 1 = yes
	**m_infec_total** = composite of fever, infection all to be used for maternal composite	Categorical<br>0 = no, 1 = yes
	**Endomet** (endometritis)	Categorical<br>0 = no, 1 = yes
	**ebl** (estimated blood loss in mL)	Continuous
Secondary maternal safety outcomes	**pph1000** (severe post-partum haemorrhage 1000ml or more)	Categorical<br>0 = no, 1 = yes
	**pph500** (post-partum haemorrhage 500ml or more)	Categorical<br>0 = no, 1 = yes
	uti (urinary tract infection)	Categorical<br>0 = no, 1 = yes
	bowel Bowel_obstruction (bowel obstruction)	Categorical<br>0 = no, 1 = yes
	vte (thromboembolic event)	Categorical<br>0 = no, 1 = yes
	hyperten hypertension (hypertensive disorder in pregnancy)	Categorical<br>0 = no, 1 = yes
	pet (pre-eclampsia/HELLP)	Categorical<br>0 = no, 1 = yes
	**icu** (maternal ICU admission)	Categorical<br>0 = no, 1 = yes
	**ut_rupture** (uterine rupture)	Categorical<br>0 = no, 1 = yes
	**m_death** (maternal death)	Categorical<br>0 = no, 1 = yes
	Other_pp (other postpartum condition requiring treatment)	Categorical<br>0 = no, 1 = yes

Secondary neonatal morbidity outcomes	**Ph_cat**<br>(arterial umbilical cord pH <7.10)	Categorical<br>0 = no (pH 7.10 or greater)<br>1 = yes (pH <7.09)
	**ph**	Continuous
	**lactate**	Categorical<br>0 = no (<4.79)<br>1 = yes (4.8 or greater)
	**nicu**<br>(admission to neonatal intensive care unit)	Categorical<br>0 = no, 1 = yes
	**seizures**<br>(neonatal seizures)	Categorical<br>0 = no, 1 = yes
	**Neo_infec_sus**<br>Infection_neonatal (suspected)	Categorical<br>0 = no, 1 = yes
	**Neo_infec_proven**<br>Infection_neonatal (proven neonatal infection)	Categorical<br>0 = no, 1 = yes
	**Neo_anbx**<br>antibiotics _neonatal (neonatal antibiotics given)	Categorical<br>0 = no, 1 = yes
	**mas**<br>(meconium aspiration syndrome)	Categorical<br>0 = no, 1 = yes
	**hie**<br>(Hypoxic ischaemic Encephalopathy)	Categorical<br>0 = no, 1 = yes
	**Stillbirth** (stillbirth)	Categorical<br>0 = no, 1 = yes
	**nnd** (neonatal death)	Categorical<br>0 = no, 1 = yes
	**Apgar**<br>APGAR score recorded	Continuous
	**Apgar_cat**<br>(APGAR score recorded as <7 at 5 minutes)	Categorical<br>0 = no (Good APGAR 7-10 at 5 min)

		1 = yes (Bad APGAR 0-6 at 5 min)
	**Neo_fever** (neonatal temperature ≥38C)	Categorical<br>0 = no, 1 = yes
	**mech_venti** (mechanical ventilation/ endotracheal intubation)	Categorical<br>0 = no, 1 = yes
	**ecc**<br>external cardiac compressions	Categorical<br>0 = no, 1 = yes
Composite outcomes	**Com_peri**<br>Composite perinatal outcome	Categorical<br>0 = no, 1 = yes
	**Com_mat**<br>Composite maternal outcome	Categorical<br>0 = no, 1 = yes
	**Com_mod**<br>Composite mode of delivery (vaginal birth)	Categorical<br>0 = caesarean, 1 = vaginal birth
	**Comp_instru**<br>Composite instrumental delivery	Categorical<br>0 = no, 1 = yes
	**Comp_cs**<br>Composite caesarean delivery	Categorical<br>0 = vaginal, 1 = caesarean
	**Comp_nvd**<br>Composite unassisted vaginal delivery	Categorical<br>0 = no (caesarean + Instrumental), 1 = yes, unassisted vaginal

Effect modifiers (treatment-covariate interaction)

Effect modifiers will be studied on the ITT population only. Only within-study interactions will be considered. We will study the following hypothesized individual-level modifiers of treatment outcome in terms of vaginal birth:

- Nulliparous or multiparous women: Parity will be recorded as such for each woman and will be analysed as a binary variable.
- Maternal age: In years with one decimal and will be analysed as a continuous variable.
- Gestational age: In weeks as integer values and will be analysed as a continuous variable.
- Maternal BMI: In kg/m2 with one decimal and will be analysed as a continuous variable.
- Initial Bishop score: Integer values and will be analysed as a continuous variable.
- Indications for IOL (if data are available enough):

Interested indications include hypertensive disorders, post-date gestation, diabetes/gestational diabetes, intrauterine growth restriction/ fetal growth restriction, and advanced maternal age. All indications will be compared to elective IOL (reference) as a categorical variable.

These interactions and their corresponding variables and possible values are summarized in the following table:

Variable	Type	Values	Description
parcode	Binary	0 = "Nulliparous"<br>1 = "Multiparous"	Nulliparous, parity=0<br>Multiparous, parity>=1
age	Continuous	One decimal	Maternal age in years
gestage	Continuous	Integer	Gestational age in weeks
bmi	Continuous	One decimal	Maternal BMI in kg/m2
bs_begi	Continuous	Integer	Bishop score at baseline
iol-ind	Categorical	0 = no, 1 = yes for the following indications separately.<br><br>Post-dates gestation<br>Hypertensive disorders<br>Diabetes/gestational diabetes<br>IUGR/fetal growth restriction<br>Advanced maternal age<br>Obstetric cholestasis<br>Oligohydramnios<br>Other<br>Elective/social	Indications for induction of labour. Other indications will not be considered.

Hypothesized interactions (effect modifiers, subgroups) that will be studied.

STATISTICAL ANALYSIS II: META-ANALYSIS

<u>Two-stage method</u>

Step 1

For each included study, the outcomes will be compared between the treatment (balloon catheter plus concurrent low-dose vaginal misoprostol) and control (low-dose vaginal misoprostol alone) groups. All models will adjust for maternal age and parity. For binary outcomes, odds ratios with 95% confidence intervals will be calculated using logistic regression. For time to vaginal delivery (induction to vaginal delivery interval), we will estimate the sub-distribution hazard ratio (sHRs) and 95% CIs using the sub-distribution hazard competing risk model, which considers caesarean section as a competing risk.

For adverse outcomes, an OR >1.0 indicates that the combined/treatment group may confer a greater risk, whereas, for positive outcomes (for example, vaginal birth), an sHR or OR >1.0 indicates that the combined/treatment group may be more favourable. Effects estimates of the above-mentioned within-study interactions will be computed for primary outcomes.

Step 2

The generated summary estimates of studies will be combined using a random-effects model, as we assume differences in treatment effect due to between-study heterogeneity.

- Heterogeneity variance τ2 will be estimated with the method of restricted maximum likelihood (REML) estimator with Hartung-Knapp-Sidik-Jonkman variance correction.
- The I_2 statistic will be calculated to provide an additional measure of between-study heterogeneity.
- 95% confidence intervals will be calculated.

- We will calculate 95% prediction intervals.
- The results will be presented in forest plots: Labour and delivery outcomes, composite maternal and neonatal outcomes will be presented in forest plots separately.

<u>One-stage method</u>

One-stage method will be the main analysis for outcomes with zero event in any group in any study (e.g., postpartum haemorrhage > 1L, uterine hyperstimulation, tachysystole, Apgar score <7 at 5 min, arterial umbilical cord pH <7·1 and admission to neonatal intensive care unit). For these outcomes, random effects logistic regression will be used. The models will have a stratified intercept and a random treatment effect, covariates including age and parity will have fixed effects. Study-specific centering will be applied to control downward bias.

STATISTICAL ANALYSIS III: SUBGROUP ANALYSIS

Subgroup effects for the primary outcomes will be assessed using interaction terms between treatment and baseline covariates. Interactions will be computed within each study and then pooled using random-effects models. This is recommended to avoid ecological bias (aggregation bias). Subgroup effects for parity (nulliparous versus multiparous), maternal age, body mass index, and gestational age at induction (as a continuous and grand-mean centered variable) will be explored.

STATISTICAL ANALYSIS III: SENSITIVITY ANALYSIS

1. ‘One-stage’ analysis for primary outcomes

Sensitivity analysis for primary outcomes using a one-stage method will be produced to see any difference between two-stage methods. We will use random effects logistic regression to allow for heterogeneity across trials. The models will have a stratified intercept and a random treatment effect. Study-specific centering and adjusting for maternal age and parity will be applied to control for downward bias.

2. IPD unavailability bias

We will extract aggregate data on vaginal birth from publications of all RCTs irrespective of data sharing status and combine these estimates in a two-stage meta-analysis. This aggregate data meta-analysis using the random-effects model (REML with H-K-S-J variance correction) will be compared to the results of the IPD meta-analysis. This method will not be possible for the two composite outcomes of maternal and neonatal safety. We will also do aggregate data meta-analysis for secondary outcomes, if the data are sufficient.

3. Publication bias

Funnel plots will be created for all eligible RCTs to see whether all RCTs fall within the expected 95% range, giving a symmetric view.

4. As-treated (AT) analysis

AT analysis for primary outcomes will be done to investigate the effect of the meta-analytical method on the pooled estimates compared to the intention-to-treat analysis.

References

1. Australian Institute of Health and Welfare. National Maternity Data Development Project: Indications for induction of labour – research brief no6 2016; PER 80
2. de Vaan MD, Ten Eikelder ML, Jozwiak M, Palmer KR, Davies-Tuck M, Bloemenkamp KW, et al. Mechanical methods for induction of labour. Cochrane Database Syst Rev. 2019;10(10):CD001233.
3. Gynecologists ACoOa. ACOG Practice Bulletin No. 107. 2009.
4. Leduc D BA, Lee L, Dy J. Induction of labour. . Journal of Obstetrics and Gynaecology Canada.2013;35(9):840-57.
5. Eser A OE, Abide C, Eser T, Eser G, Abike F et al. Transcervical Foley balloon catheter and vaginal prostaglandin E2 insert combination vs. vaginal prostaglandin E2 insert only for induction of labor at term: a randomized clinical trial. Archives of Gynecology and Obstetrics 2018;2018;299(2):451-457.
6. L G. Methods of Cervical Ripening and Labor Induction. Clinical Obstetrics and Gynecology.2014;57(2):377-390.

7. AC S. Methods of Cervical Ripening and Labor Induction: Mechanical. 2014;2014;57(2):369-76.
8. Stewart LA TJ. To IPD or not to IPD? . Evaluation & the Health Professions.2002;25(1):76-97.
9. Stewart LAea. 'Preferred Reporting Items for Systematic Review and Meta-Analyses of individual participant data: the PRISMA-IPD Statement.'. JAMA. 2015;313(16), pp. 1657–65.
10. Sterne JACea. 'RoB 2: A revised tool for assessing risk of bias in randomised trials'. The BMJ. 2019(366):1-8.
11. Mol BW, Lai S, Rahim A, Bordewijk EM, Wang R, van ER, et al. Checklist to assess Trustworthiness in RAndomised Controlled Trials (TRACT checklist): concept proposal and pilot. Res Integr Peer Rev. 2023;8(1):6.
