# Supplementary material for: Balloon catheter plus concurrent vaginal misoprostol versus vaginal misoprostol alone for cervical ripening in induction of labor: A systematic review and an individual participant data meta‐analysis of randomized controlled trials
Source: Int J Gynaecol Obstet. 2026 Mar 12;174(3):1188–204. doi: 10.1002/ijgo.70949 (PMC13505249; doi:10.1002/ijgo.70949)
Supplement: Supplementary file 1 [file IJGO-174-1188-s001.zip › Appendix B - Data Dictionary (Data code).docx]

**IPD-MA data dictionary for** *“*Balloon catheter plus concurrent low-dose vaginal misoprostol versus low-dose vaginal misoprostol alone for induction of labour: individual participant data meta-analysis of randomised controlled trials”

**Intervention (Group A)** - balloon catheter plus concurrent low-dose vaginal misoprostol.

**Reference/Control (Group B) -** vaginal misoprostol.

| Identification | **my_ID**  (my participant ID) | Continuous |
| --- | --- | --- |
|  | **Original_ID**  (old participant ID) | Continuous  Study ID used in the original study |
|  | **author** | RCT name_year |
|  | **RCT** | Continuous  1=  2=  3=  4=  5=  6=  7=  8= |
| Induction category | **ind_cat**  induction category | Categorical  0 = vaginal misoprostol  1 = combined |
|  | **Singleton**  Fetal number | Categorical  0 = singleton, 1 = multiple |
| Baseline variables | **Age** years | Continuous |
|  | **Weight** /kg | Continuous |
|  | **Height**/cm | Continuous |
|  | **bmi** kg/m2 | Continuous |
|  | **gesta_weeks**  (gestational age in weeks) | Continuous |
|  | ethnicity | Categorical  0 = Caucasian  1 = Hispanic  2 = Asian  3 = Black  4 = other (specify) |
|  | smoking  (Smoking status) | Categorical  0 = no, 1 = yes |
|  | **parity** | Continuous |
|  | **parcode**  (parity code) | Categorical  0 = nulliparous  1 = multiparous |
|  | **Ind_iol** (indication for IOL) | |
|  | Categorical  0 = other/unknown  1 = hypertensive disorders  2 = postdates  3 = diabetes/gestational diabetes  4 = IUGR/fetal growth restriction  5 = oligohydramnios  6 = advanced maternal age  7 = obstetric cholestasis  8 = elective/social  9 = >1 indication | |
| **bs_initial**  (initial Bishop score before intervention) | | Continuous |
| **bs_max**  (Maximum Bishop score achieved) | | Continuous |
| **srom**  Membrane status | | Categorical  1= intact  0= ruptured |
| Primary and secondary delivery outcomes | **mod**  (mode of delivery) | Categorical  2 = instrumental  1 = caesarean section  0 = vaginal unassisted |
|  | **Instr_ftp**  Instrumental delivery for failure to progress | Categorical  0 = no, 1 = yes |
|  | **Instr_fhr**  instrumental delivery for abnormal FHR | Categorical  0 = no, 1 = yes |
|  | **Cs_ftp_new**  Caesarean section for failure to progress | Categorical  0 = no, 1 = yes |
|  | **Cs_abn_ftr**  Caesarean section for abnormal FHR | Categorical  0 = no, 1 = yes |
|  | **other_methods** (use of other methods for IOL after originally allocated method) | Categorical  0 = no, 1 = yes |
|  | **Idi_hr** (induction to delivery interval in hours) | Continuous |
|  | **Idi_min** (induction to delivery interval in min) | Continuous |
|  | **ut_tachy**  (uterine tachysystole | Categorical  0 = no, 1 = yes |
|  | **ut_ hypertonus**  hypertonus | Categorical  0 = no, 1 = yes |
|  | **ut_hypersti**  (uterine hyperstimulation) | Categorical  0 = no, 1 = yes |
|  | Oxy_aug  (use of oxytocin during labour) | Categorical  0 = no, 1 = yes |
| Secondary labour progression outcomes | **Mec_amnio**  (meconium stained  amniotic fluid ) | Categorical  0 = no, 1 = yes |
|  | **analge**  (analgesia during labour) | Categorical  0 = no, 1 = yes |
|  | **epidural**  Epidural use | Categorical  0 = no, 1 = yes |
|  | **analge_cat**  type_analgesia  (type of analgesia during labour) | Categorial  0 = None  1 = Parental opioids  2 = Epidural use  3 = Entonox  4 = Other |
|  | **oxy_duration** (duration of oxytocin infusion in hours) | Continuous |
|  | **m_anbx**  (maternal antibiotics given) | Categorical  0 = no, 1 = yes |
|  | **m_fever** (maternal temperature ≥38C) | Categorical  0 = no, 1 = yes |
|  | **m_infec**  (suspected/proven maternal infection) | Categorical  0 = no, 1 = yes |
|  | **m_infec_total** = composite of fever, infection all to be used for maternal composite | Categorical  0 = no, 1 = yes |
|  | **Endomet**  (endometritis) | Categorical  0 = no, 1 = yes |
|  | **ebl** (estimated blood loss in mL) | Continuous |
| Secondary maternal safety outcomes | **pph1000**  (severe post-partum haemorrhage 1000ml or more) | Categorical  0 = no, 1 = yes |
|  | **pph500**  (post-partum haemorrhage 500ml or more) | Categorical  0 = no, 1 = yes |
|  | **uti**  (urinary tract infection) | Categorical  0 = no, 1 = yes |
|  | **bowel**  Bowel_obstruction  (bowel obstruction) | Categorical  0 = no, 1 = yes |
|  | **vte**  (thromboembolic event) | Categorical  0 = no, 1 = yes |
|  | **hyperten**  hypertension  (hypertensive disorder in pregnancy) | Categorical  0 = no, 1 = yes |
|  | **pet**  (pre-eclampsia/HELLP) | Categorical  0 = no, 1 = yes |
|  | **icu**  (maternal ICU admission) | Categorical  0 = no, 1 = yes |
|  | **ut_rupture** (uterine rupture) | Categorical  0 = no, 1 = yes |
|  | **m_death**  (maternal death) | Categorical  0 = no, 1 = yes |
|  | Other_pp  (other postpartum condition requiring treatment) | Categorical  0 = no, 1 = yes |
| Secondary neonatal morbidity outcomes | **ph_cat**  (arterial umbilical cord pH <7.10) | Categorical  0 = no (pH 7.10 or greater)  1 = yes (pH <7.09) |
|  | **ph** | Continuous |
|  | **lactate** | Categorical  0 = no (<4.79)  1 = yes (4.8 or greater) |
|  | **nicu**  (admission to neonatal intensive care unit) | Categorical  0 = no, 1 = yes |
|  | **seizures**  (neonatal seizures) | Categorical  0 = no, 1 = yes |
|  | **neo_infec_sus**  Infection_neonatal  (suspected) | Categorical  0 = no, 1 = yes |
|  | **neo_infec_proven**  Infection_neonatal  (proven neonatal infection) | Categorical  0 = no, 1 = yes |
|  | **neo_anbx**  antibiotics _neonatal  (neonatal antibiotics given) | Categorical  0 = no, 1 = yes |
|  | **mas**  (meconium aspiration syndrome) | Categorical  0 = no, 1 = yes |
|  | **hie**  (Hypoxic ischaemic Encephalopathy) | Categorical  0 = no, 1 = yes |
|  | **stillbirth** (stillbirth) | Categorical  0 = no, 1 = yes |
|  | **nnd** (neonatal death) | Categorical  0 = no, 1 = yes |
|  | **apgar**  APGAR score recorded | Continuous |
|  | **apgar_cat**  (APGAR score recorded as <7 at 5 minutes) | Categorical  0 = no (Good APGAR 7-10 at 5 min)  1 = yes (Bad APGAR 0-6 at 5 min) |
|  | **neo_fever** (neonatal temperature ≥38C) | Categorical  0 = no, 1 = yes |
|  | **mech_venti**  (mechanical ventilation/ endotracheal intubation) | Categorical  0 = no, 1 = yes |
|  | **ecc**  external cardiac compressions | Categorical  0 = no, 1 = yes |
| Composite outcomes | **com_peri**  Composite perinatal outcome | Categorical  0 = no, 1 = yes |
|  | **com_mat**  Composite maternal outcome | Categorical  0 = no, 1 = yes |
|  | **com_mod**  Composite mode of delivery (vaginal birth) | Categorical  0 = caesarean, 1 = vaginal birth |
|  | **comp_instru**  Composite instrumental delivery | Categorical  0 = no, 1 = yes |
|  | **comp_cs**  Composite caesarean delivery | Categorical  0 = vaginal, 1 = caesarean |
|  | **comp_nvd**  Composite unassisted vaginal delivery | Categorical  0 = no (caesarean + Instrumental), 1 = yes, unassisted vaginal |
