# Supplementary material for: Balloon catheter plus concurrent vaginal misoprostol versus vaginal misoprostol alone for cervical ripening in induction of labor: A systematic review and an individual participant data meta‐analysis of randomized controlled trials
Source: Int J Gynaecol Obstet. 2026 Mar 12;174(3):1188–204. doi: 10.1002/ijgo.70949 (PMC13505249; doi:10.1002/ijgo.70949)
Supplement: Supplementary file 1 [file IJGO-174-1188-s001.zip › Appendix C - TRACT Checklist.pdf]

Appendix 2

The TRACT Screening Checklist

Overview

This screening tool aims to help identify and triage studies at risk of integrity issues. The checklist includes eight domains which are applicable to every RCT; governance, author group, plausibility of intervention usage, timeframe, drop-out rates, baseline characteristics and outcomes. The tool is a proposal to optimise our awareness of research integrity and has not yet been validated.

Users

This tool is designed to be used by clinical experts on articles in their field of study as a degree of clinical judgement and experience will be required for some items, and especially those using subjective or descriptive terms. It may also be beneficial to seek assistance from a statistician for some items.

Instructions for Use

The screening tool requires information found in the full text of the trial report and trial registration (if applicable) – please ensure you collect these prior to using the tool. Each item in the checklist is rated using a colour-coded system: green suggests 'No Concerns', yellow suggests 'Some Concern/No Information', and red suggests 'Major Concern'. For each item, the user should choose one rating and address the rationale for the chosen rating in the 'Support of Judgement' section. There is also a free-text space for users to add additional comments about other integrity issues if required.

Details

Article Title, Year	
Author(s)	

Checklist

DOMAIN	*ITEM*	*RATING*			*SUPPORT FOR JUDGEMENT*
		No Concerns	Some Concerns/ No Information	Major Concerns	
1. Governance	Absent or retrospective registration of RCTs. This is relevant for RCTs commencing after 2010				
	Discrepancy of >15% between the intended sample size in the trial registration compared to the actual sample size achieved in the RCT				
	Absent or vague description of research ethics or apparent concerns regarding ethics				
2. Author Group	Number of authors ≤3 or low author to study size ratio				
	Other studies of authors have				

	been retracted not on request of the authors				
	Large number of RCTs published in a short time frame by one author/in one institute				
3. Plausibility of Intervention Usage	Insufficient or implausible description of allocation concealment (e.g. two interventions but only one placebo)				
	Unnecessary or illogical description of methodological standards (e.g. use of sealed envelopes in a placebo-controlled trial)				
4. Timeframe	Fast recruitment of participants within the study time (especially single centre studies)				
	Short or impossible time frame between ending recruitment/follow up and submission of the paper (take into account time to outcome e.g. live birth, pregnancy outcome etc.)				
5. Drop-Out Rates	Zero participants lost to follow up or no reasons mentioned for loss of follow up				
	Ideal number of losses to follow up resulting in perfectly rounded number in each group (e.g. groups of 50 or 100)				
6. Baseline Characteristics	No or few baseline (<5) characteristics presented				
	Implausible patient characteristics judging from common sense, the literature and local data (e.g. similar standard deviations for completely different characteristics with different means and distributions)				
	Perfect balance for multiple baseline characteristics or significant/large differences between baseline characteristics				
	Important prognostic factors are not reported as baseline characteristics				
7. Outcomes	Effect size that is much larger than in other RCTs regarding the same topic				
	Conflicting information between outcomes (e.g. more ongoing				

	pregnancies than clinical pregnancies)				
	Change in primary outcome from registration to publication				

Additional Comments
